# Bias Due to Within-Subject Exposure Dependency With or Without Bias Due to Lack of Pairwise Exchangeability When Exposure Is Chronic in Case-Crossover and Case–Time-Control Studies: A Simulation Study

**DOI:** 10.1093/aje/kwad104

**Published:** 2023-04-21

**Authors:** Kiyoshi Kubota, Thu-Lan Kelly

**Keywords:** bias, case-crossover studies, case–time-control studies, self-controlled studies

## Abstract

The case-crossover study design has been proposed as a suitable design for use when a brief exposure causes a transient change in risk of an acute-onset disease. In pharmacoepidemiology, the condition of “brief exposure” is rarely satisfied because medication use is often chronic or successive, which may result in bias due to within-subject exposure dependency. Here we describe a simulation of a case-crossover study conducted within a cohort, where patients successively used a drug for 60 or more days and the rate ratio for the outcome occurrence was 4.0. Standard conditional logistic regression for the analysis produced overestimated odds ratios ranging up to 7.8. This bias due to within-subject exposure dependency from chronic use can be removed by the Mantel-Haenszel method or by our recently proposed weighting method. We also show that when some patients are censored after switching to another drug, a lack of pairwise exchangeability causes bias which is similar to bias due to an exposure time trend. This bias can be removed by using the case–time-control study design. We show that bias due to within-subject exposure dependency and lack of pairwise exchangeability occur independently and can occur separately or simultaneously, and we demonstrate how to detect and remove them.

## Abbreviations

CI: confidence interval
OR: odds ratio
RR: rate ratio

The case-crossover study design is a case-only design which exclusively uses data from subjects who have experienced an outcome of interest. It was originally proposed as a design suitable for instances where a brief or intermittent exposure causes a transient change in risk of a rare acute-onset disease (1, 2). The original unidirectional case-crossover study included discordant cases who had both exposed and unexposed periods at and before the outcome occurrence and did not require data collected after the outcome. This is an advantage where the outcome affects the future exposure probability or future observation periods, which is difficult to manage using other case-only designs, including self-controlled case series (3, 4). For example, an occurrence of an adverse reaction to a medication may affect future use of the drug. On the other hand, the requirement of brief exposure is rarely satisfied in some fields like pharmacoepidemiology (5, 6), where medication use is often chronic or successive and exposure is not independent between periods. In 2001, Vines and Farrington (7) indicated that analysis using standard conditional logistic regression may lead to biased results in case-crossover studies when within-subject exposure dependency or autocorrelation exists even when the exposure time trends do not. Recently, we proposed a novel weighting method (8) by extending the findings of Vines and Farrington and modifying the likelihood presented by Greenland (9). Our weighting method for removing bias due to within-subject exposure dependency can be used with or without a time-varying confounder (8).

One of other biases in case-crossover studies is due to exposure time trends associated with the increase or decrease of the proportion exposed in the population, which can be removed using the case–time-control method (10). Recently, Bykov et al. (11) indicated that persistent user bias due to persistent users is also removed by the case–time-control approach.

The case-crossover analysis involving chronic drug use has often yielded complicated results that are difficult to explain. For example, Delaney and Suissa (12) used a case-crossover design to investigate the association between warfarin use and bleeding to show how the case-crossover design in pharmacoepidemiology is vulnerable to design parameters. When exposure was defined as daily use within the previous month, there was no association and the rate ratio (RR) was 0.98 (95% confidence interval (CI): 0.74, 1.28), but when subjects were stratified by the intermittency of warfarin therapy, the RR was 2.59 (95% CI: 1.42, 4.74) in a stratum of transient exposure, defined as 1–3 prescriptions in the previous year (12). When the exposure time window was increased to 1 year, the RR was 3.57 (95% CI: 2.22, 5.05), which was reduced by the case–time-control study design to 1.72 (95% CI: 1.08, 2.43). The authors highlighted that “the design is… highly sensitive to assumptions about intermittency of drug use and the length of the exposure time window” (12, p. 53).

To contextualize the problems encountered in the real-world data associated with chronic medication use, we have conducted a simulation study by which bias due to within-subject exposure dependency and exposure time trends can be addressed separately from biases due to a change in patients’ susceptibility to the outcome occurrence, or other changes in the drug effect (e.g., tolerance development) associated with chronic use of a drug. In our current simulation study, we show that bias due to within-subject exposure dependency associated with chronic or successive exposure can be removed by means of the Mantel-Haenszel method or our recently proposed weighting method. We also show that bias takes place when a proportion of patients are censored due to switching to another drug, and this bias can be removed by the case–time-control method (see the Web Appendix and Web Figures 1–3, available at https://doi.org/10.1093/aje/kwad104, for illustrative examples of those biases).

By using the approach illustrated in this study, the requirement of brief exposure may be relaxed and the case-crossover study design may be used reliably, together with the case–time-control approach when needed, even if the exposure is chronic or successive.

## METHODS

### Data

**Figure 1:**
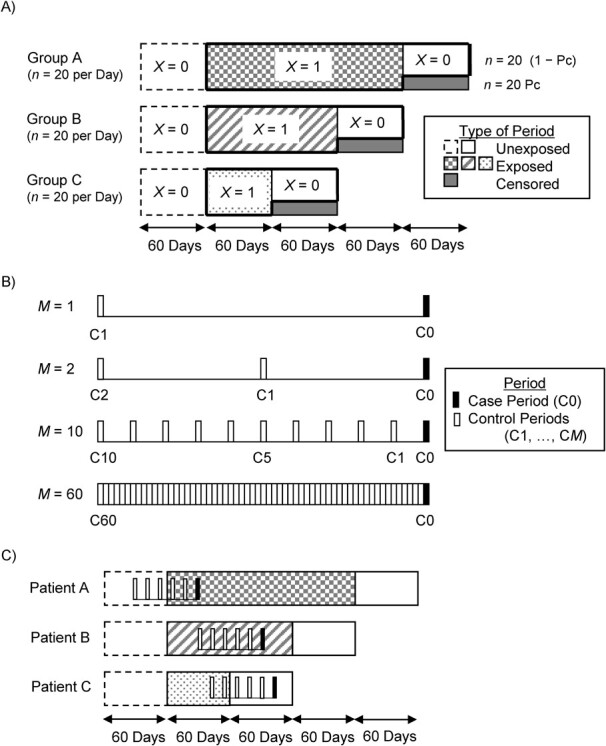
Design diagrams for a simulation of case-crossover studies conducted within a cohort. A) Distribution of 60 patients who start using a medication and enter a cohort study every day after 60 days of the unexposed period. Of the 60 patients, groups A, B, and C (*n* = 20 each) use the drug for 180, 120, and 60 days, respectively. After the exposed period ends, 20 (1 − Pc) patients become unexposed, while 20 Pc patients switch to another drug and are censored, where Pc represents the proportion of censored patients. B) Case-crossover studies conducted within the cohort study, where C0 denotes the case period and C1, C2, …, C*M* denote control periods and *M* is the number of control periods. C) Distribution of 3 patients (patients A, B, and C) who had an outcome while followed in the cohort study and were included in the case-crossover study with 1 case period and 5 control periods. Patient A, who had an outcome during the first 60-day exposed period, had the discordant exposure pattern and was included in the case-crossover analysis; patient B, who had an outcome after the first 60 days of the exposed period, had the concordant exposure pattern (always exposed) and was excluded from the analysis; and patient C, who had an outcome during the 60-day postexposure period, had the discordant exposure pattern and was included in the analysis.

We simulated data for a cohort study in which 1 period was 1 day and exposure to a drug occurred successively (Figure 1). We assumed that the effect of a drug was acute and disappeared completely 24 hours after a patient took the drug once daily. After a preexposure (washout) period of 60 or more days when patients did not use the drug and did not have an outcome of interest, we assumed that 60 patients started a drug of interest and entered the cohort every day and that all patients continued to use the drug for at least 60 successive days. To simplify scenarios, we subdivided the 60 patients into 3 groups (groups A, B, and C) who used the drug for 180, 120, and 60 successive days, respectively. After the end of the exposure period, some patients stopped using the drug and were followed until the end of a 60-day postexposure (unexposed) period, while the other patients switched to another drug and were censored immediately after the exposure period ended. In the context of the cohort study, patients who were followed until the 60-day postexposure period had exposed periods followed by unexposed periods, while patients who were censored after switching to another drug had exposed periods only.

In scenarios 1 and 2, we assumed that the rate of occurrence of the outcome was 0.00005 events per day (0.018 per year) during the unexposed postexposure period and that the rate was 0.0002 events per day (0.073 per year) during the exposure period, so that the RR was 4.0. As a reference, in scenarios 3 and 4, we also generated data for a scenario in which the rate was 0.00005 per day during the exposed period (i.e., RR = 1.0). We simulated data for scenarios where 0% (scenarios 1 and 3) and 30% (scenarios 2 and 4) of patients were censored after switching. We also simulated extra scenarios in which the incidence rates were one tenth (0.000005 per day) and one hundredth (0.0000005 per day), but we do not report the results because the conclusion was the same as in the current study.

We conducted case-crossover and case–time-control studies by selecting subjects from the cohort study. Cases for a unidirectional case-crossover study were identified during a study period of 200 days when a steady state was attained in the cohort—that is, when the size of the population and the proportion of those exposed did not change even if some patients entered and left the cohort every day (see Web Figure 4). If a patient had 2 or more outcome occurrences, only the first occurrence was included in the case-crossover analysis. The time window for the case-crossover study was 61 days in and before the case period, and all time periods were 1 day. The number of control periods (*M*) was set as 1, 2, 3, 4, 5, 6, 10, 12, 15, 20, 30, and 60. When *M* = 1, a control period was 60 days prior to the case period; when *M* = 2, two control periods were 30 and 60 days prior to the case period. In general, *M* control periods were 60/*M*, 2 × 60/*M*, …, *M* × 60/*M* days before the case period (see Figure 1B). We selected patients with a discordant exposure pattern because only those patients contributed to a case-crossover analysis (see patient A or patient C in Figure 1C). Cases who had the outcome during the exposure period but after the first 60 days with a concordant exposure pattern were excluded (see patient B in Figure 1C). Patients who had the outcome after switching to another drug were not identified as cases and not included in the analysis because they were censored before the outcome occurred.

In the case–time-control study, risk set sampling was used to select 1 control subject (designated the “time-control” by Greenland (9)) per case. A time-control was randomly selected with replacement from those who were at risk of becoming a case and had a discordant exposure pattern during the selected time period. As a sensitivity analysis, we also randomly selected time-controls irrespective of whether they had a discordant or concordant exposure pattern.

### Analyses

We estimated odds ratios (ORs) via 3 methods: standard conditional logistic regression (OR_SCL_), the Mantel-Haenszel method (OR_MH_), and our weighting method (modified Greenland’s likelihood) OR_G_ (8). The following likelihood was used to estimate OR_G_ and OR_SCL_:

(1) \begin{equation*} L=\prod \limits_{i=1}^N\frac{\exp \left(\left(d\mathrm{\beta} +\mathrm{\theta} \right){x}_{i0}\right){w}_{i0}}{\sum_{j=0}^M\exp \left(\left(d\mathrm{\beta} +\mathrm{\theta} \right){x}_{ij}\right){w}_{ij}}, \end{equation*}

where *N* is the total number of cases and time-controls, *d* = 1 for cases while *d* = 0 for time-controls, and ${x}_{ij}$ and ${w}_{ij}$ are the status of the exposure (and time-varying confounder) and weight for the *j*th period (*j* = 0 for a case period and *j* = 1, 2, …, *M* for *M* control periods) in patient *i*, respectively. The weight ${w}_{ij}$ is set to 1 to estimate OR_SCL_. To estimate OR_G_, we first define ${m}_i^1$ and ${m}_i^0$ as the number of exposed periods and the number of unexposed periods (including both case and control periods) of patient *i*, respectively (${m}_i^1+{m}_i^0=M+1$). Then the weight ${w}_{ij}$ with which to estimate OR_G_ is defined as ${w}_{ij}={\pi}_{10}/{m}_i^1$ when ${x}_{ij}=1$ and ${w}_{ij}=1/{m}_i^0$ when ${x}_{ij}=0$. The quantity ${\mathrm{\pi}}_{10}$ is estimated as in the previous study (8) as follows:

(2) \begin{equation*} {\mathrm{\pi}}_{10}=\frac{\sum_i{\textrm{PT}}_{01i}/\left({a}_0+{b}_0\right)\ }{\sum_i{\textrm{PT}}_{10i}/\left({a}_1+{b}_1\right)\ }. \end{equation*}

In equation 2, PT represents the patient-time and ${\textrm{PT}}_{10i}$ is the number of unexposed control periods for subject *i* who is a case or time-control with the exposed case period; ${\textrm{PT}}_{01i}$ is the number of exposed control periods for subject *i* with the unexposed case period; ${a}_0$ and ${b}_0$ are the numbers of cases and time-controls with an unexposed case period and at least 1 exposed control period, respectively; and ${a}_1$ and ${b}_1$ are the numbers of cases and time-controls with an exposed case period and at least 1 unexposed control period, respectively (see Web Figure 5 for estimation of weights).

Pairwise exchangeability is the relationship $P({X}_{i0}=1, {X}_{im}=0)=P\left({X}_{i0}=0,{X}_{im}=1\right)$, where ${X}_{i0}$ is the exposure status in the case period and ${X}_{im}$ is the exposure status in the *m*th control period preceding the case period (when $t=-m$) in patient *i* (7). When pairwise exchangeability holds for all values of *m* (*m* = 1, 2, …, *M*), the estimate of $\exp \left(\mathrm{\theta} \right)$ is equal to 1 and an $\exp \left(\mathrm{\theta} \right)$ that is not equal to 1 indicates the presence of bias due to lack of pairwise exchangeability.

In a case-crossover study with no time-controls, $\left(d\mathrm{\beta} +\mathrm{\theta} \right)$ in equation 1 is replaced by $\mathrm{\beta}$; ${b}_0$ and ${b}_1$ in equation 2 are 0; and the numbers of exposed and unexposed periods are calculated from the cases only.

In the case–time-control approach, the OR from the Mantel-Haenszel method, OR_MH_, is estimated as OR_MH_ = OR_MH_case_/OR_MH_tc_, where OR_MH_case_ is estimated from cases and OR_MH_tc_ is estimated from time-controls.

Estimates and 95% CIs for OR_SCL_, OR_MH_, and OR_G_ were calculated from the mean and standard deviation of the logarithm of estimates from each of 1,000 iterations of the simulation. SAS, version 9.4 (SAS Institute, Inc., Cary, North Carolina), was used for all analyses.

## RESULTS

**Figure 2:**
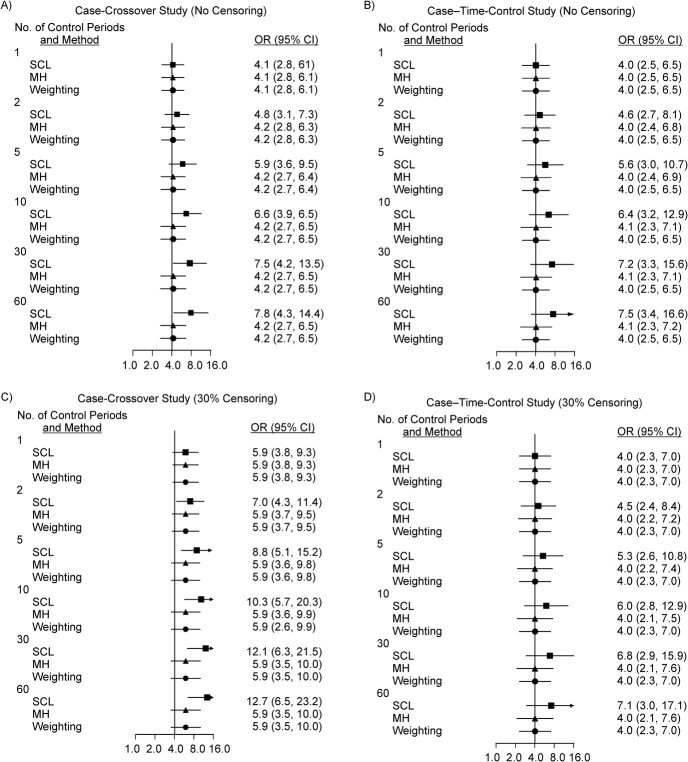
Odds ratios (ORs; points) and 95% confidence intervals (CIs; bars) estimated in case-crossover and case–time-control studies in scenarios 1 and 2 (true rate ratio = 4). ORs estimated by standard conditional logistic regression (SCL; ■), by the Mantel-Haenszel method (MH; ▲), and by the weighting method (weighting; 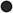) are shown for *M* = 1, 2, 5, 10, 30, and 60 control periods. Panels A and B show ORs estimated in case-crossover studies (A) and case–time-control studies (B) for scenario 1, where no patients are censored at the end of the exposed period. Panels C and D show ORs estimated in case-crossover studies (C) and case–time-control studies (D) for scenario 2, where 30% of patients are censored.

Figures and tables presenting the results are listed in Web Table 1. Figure 2 shows OR_SCL_, OR_MH_, and OR_G_ for selected values of *M*; Web Table 2 shows those estimates for all values of *M* for scenario 1, where RR = 4 and no patients were censored due to switching to another drug (Figures 2A and 2B), and for scenario 2, where RR = 4 and 30% of patients were censored (Figures 2C and 2D). When no patients were censored, OR_SCL_ for the case-crossover study was not biased when *M* = 1, but it increased to 7.8 when *M* = 60. In contrast, OR_MH_ and OR_G_ were unbiased irrespective of the value of *M* (Figure 2A). With the case–time-control approach, OR_SCL_, OR_MH_, and OR_G_ were essentially the same as those in the case-crossover study (Figure 2B). When RR = 4 and 30% of patients were censored, OR_SCL_, OR_MH_, and OR_G_ were overestimated as 5.9 when *M* = 1 in the case-crossover study. OR_SCL_ increased further to 12.7 when *M* = 60, while OR_MH_ and OR_G_ were stable but remained overestimated as 5.9 (Figure 2C). With the case–time-control design, OR_SCL_ was unbiased when *M* = 1 but increased when *M* increased, while OR_MH_ and OR_G_ were unbiased (Figure 2D). In the case–time-control studies where time-controls were selected irrespective of whether they had a discordant or concordant exposure pattern, the point estimates of the ORs were the same as those in Figures 2B and 2D, but the 95% CIs were wider because time-controls with a concordant exposure pattern were excluded from the analysis (data not shown).

**Figure 3:**
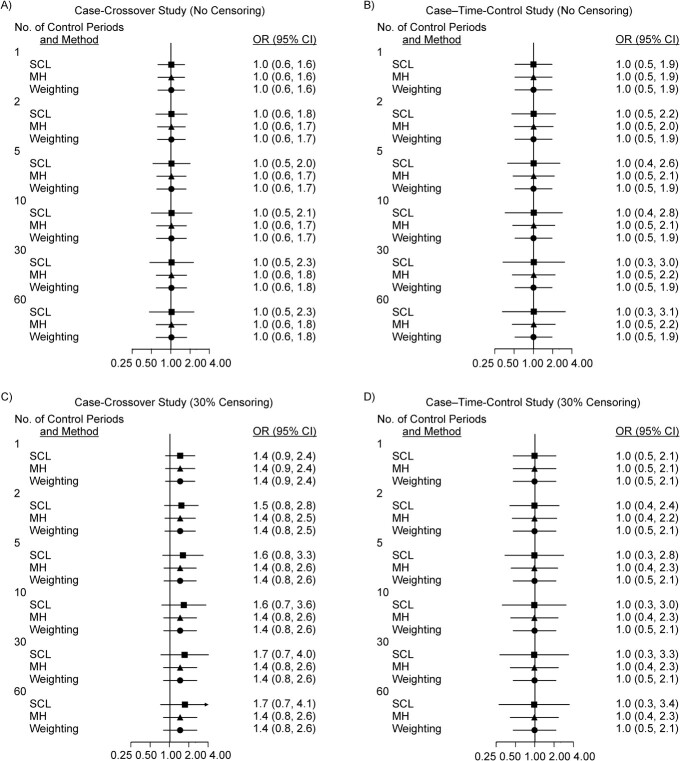
Odds ratios (ORs; points) and 95% confidence intervals (CIs; bars) estimated in case-crossover and case–time-control studies in scenarios 3 and 4 (true rate ratio  = 1). ORs estimated by standard conditional logistic regression (SCL; ■), by the Mantel-Haenszel method (MH; ▲), and by the weighting method (weighting; 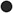) are shown for *M* = 1, 2, 5, 10, 30, and 60 control periods. Panels A and B show ORs estimated in case-crossover studies (A) and case–time-control studies (B) for scenario 3, where no patients are censored at the end of the exposed period. Panels C and D show ORs estimated in case-crossover studies (C) and case–time-control studies (D) for scenario 4, where 30% of patients are censored.

Figure 3 shows OR_SCL_, OR_MH_, and OR_G_ for scenario 3, where RR = 1 and no patient was censored (Figures 3A and 3B), and for scenario 4, where RR = 1 and 30% of patients were censored (Figures 3C and 3D). In scenario 3, where no patients were censored, OR_SCL_, OR_MH_, and OR_G_ were all unbiased in the case-crossover study, even when *M* varied and did not change when the case–time-control design was used (Figures 3A and 3B). In scenario 4, where RR = 1 and 30% of patients were censored, in the case-crossover study OR_SCL_, OR_MH_, and OR_G_ were all equal to 1.4 when *M* = 1. OR_SCL_ increased slightly to 1.7 when *M* = 60, but OR_MH_ and OR_G_ were stable at approximately 1.4 (Figure 3C). With the case–time-control approach, OR_SCL_, OR_MH_, and OR_G_ were all unbiased (Figure 3D).

As shown in Web Tables 2–5, in the case–time-control studies, OR_SCL_ and OR_G_ for time-controls were approximately 1.0 when there was no censoring (scenario 1 in Web Table 2 and scenario 3 in Web Table 4) but were larger than 1.5 with 30% censoring (scenario 2 in Web Table 3 and scenario 4 in Web Table 5). This indicates that pairwise exchangeability holds when there is no censoring but does not hold with censoring.

Table 1 shows the biases due to 1) within-subject exposure dependency and 2) lack of pairwise exchangeability and how the measures against these biases work. When both biases do not exist, OR_SCL_ is unbiased (Table 1, rows A and E). When bias due to lack of pairwise exchangeability does not exist but bias due to within-subject exposure dependency exists, OR_SCL_ is overestimated when RR = 4, but the Mantel-Haenszel method or weighting method removes bias while the case–time-control approach does not (Table 1, row B). This bias does not occur when RR = 1 (Table 1, row F). When bias due to lack of pairwise exchangeability exists but bias due to within-subject exposure dependency does not, OR_SCL_ is overestimated irrespective of RR = 4 or RR = 1, and this bias can be removed using the case–time-control approach but not by the Mantel-Haenszel method or the weighting method (Table 1, rows C and G). If 2 types of biases occur at the same time, the simultaneous use of the Mantel-Haenszel or weighting method and the case–time-control approach removes bias, but using only 1 of these 2 remedies does not when RR = 4 (Table 1, row D). However, when RR = 1, the use of the case–time-control approach is enough to remove bias (Table 1, row H).

**Table 1:** Values of Log(OR/RR) Needed to Indicate the Magnitude of the Bias of the OR Classified by the True RR, Bias Due to Lack of Pairwise Exchangeability, and Bias Due to Within-Subject Exposure Dependency in Case-Crossover and Case–Time-Control Studies

				**Case-Crossover Study**			**Case–Time-Control Study**		
**Scenario **	**Example**	**Bias Due to Lack of Pairwise Exchangeability**	**Bias Due to Within-Subject Exposure Dependency**	**OR**_**SCL**_ ^**a**^	**OR**_**MH**_ ^**b**^	**OR**_**G**_ ^**c**^	**OR**_**SCL**_	**OR**_**MH**_	**OR**_**G**_
Scenario 1									
A	RR = 4.0, no censoring, and *M* = 1^d^	No	No	0.032	0.032	0.032	0.004	0.004	0.004
B	RR = 4.0, no censoring, and *M* = 60	No	Yes	0.672^e^	0.044	0.044	0.629^e^	0.015	0.004
Scenario 2									
C	RR = 4.0, 30% censoring, and *M* = 1	Yes	No	0.390^e^	0.390^e^	0.390^e^	0.006	0.006	0.006
D	RR = 4.0, 30% censoring, and *M* = 60	Yes	Yes	1.159^e^	0.396^e^	0.396^e^	0.579^e^	0.002	0.005
Scenario 3									
E	RR = 1.0, no censoring, and *M* = 1	No	No	0.013	0.013	0.013	0.000	0.000	0.000
F	RR = 1.0, no censoring, and *M* = 60	No	Yes	0.026	0.019	0.019	0.010	0.008	0.000
Scenario 4									
G	RR = 1.0, 30% censoring, and *M* = 1	Yes	No	0.369^e^	0.369^e^	0.369^e^	−0.015	−0.015	−0.015
H	RR = 1.0, 30% censoring, and *M* = 60	Yes	Yes	0.540^e^	0.368^e^	0.368^e^	−0.029	−0.022	−0.015

Abbreviations: OR, odds ratio; RR, rate ratio.

^a^ OR estimated by standard conditional logistic regression.

^b^ OR estimated by the Mantel-Haenszel method.

^c^ OR estimated by our weighting method (modified Greenland’s likelihood) (8).

^d^ *M* represents the number of control periods.

^e^ The absolute value was more than 0.05 (about 5% difference from the true value). Log(OR/RR) ≤0.05 indicates minor bias, while log(OR/RR) >0.05 indicates major bias.

###  

While both the weighting and Mantel-Haenszel methods can reduce bias due to within-subject exposure dependency, the weighting method has an advantage when there is a time-varying confounder, which can be included as a covariate in the conditional regression model (8). In the Mantel-Haenszel method, the binary time-varying confounder is used as a stratification variable as described in the original proposal of the case-crossover study by Maclure (1). To estimate OR_MH_, cases are excluded if the status of the time-varying confounder in all control periods is different from the case period. Otherwise, cases are not excluded, but only control periods with the same status of a time-varying confounder as the case period are used in estimating OR_MH_.

Similarly to the previous scenarios 1–4, we simulated data where the incidence rate = 0.00005 events per day when unexposed and without confounding and RR = 4.0. We included a binary time-varying confounder *z*, with rate ratio RR*_z_* = 2.0. We assumed that the proportion of the time-varying confounder was 0.2 during unexposed periods (*f*_0_) and 0.4 during exposed periods (*f*_1_), and that in any period the time-varying confounder was independent of the exposure and time-varying confounder in different periods. Censoring after switching to another drug was 0% and 30% (scenarios 5 and 6, respectively). We also simulated data where RR = 1.0, RR*_z_* = 2.0, and 0% and 30% of patients were censored after switching (scenarios 7 and 8, respectively).

Simulation results are shown in Web Figures 6 and 7 and Web Tables 6–9. The estimates for the time-varying confounder were unbiased (OR = 2.0), and the OR for the time-varying confounder in time-controls was always 1.0 for scenarios 5–8. Regarding the ORs for exposure, the existence of biases and the effects of the methods for removing them (such as the Mantel-Haenszel method, the weighting method, or the case–time-control approach) were essentially the same as in the scenarios without the time-varying confounder, although the magnitude of the bias differed: For example, when RR = 4 and there was no censoring, OR_SCL_ = 7.8 without the time-varying confounder (scenario 1, Figure 2A, and Web Table 1) but OR_SCL_ = 8.3 with the time-varying confounder (scenario 5, Web Table 5). Likewise, OR_MH_ and OR_G_ were generally close to each other. However, OR_G_ was more precise and stable than OR_MH_ because all case and control periods were used to estimate OR_G_, while for estimation of OR_MH_ some cases and control periods were excluded.

As Table 2 shows, when a time-varying confounder did not exist, when RR = 4, when *M* = 1, and when no censoring occurred (scenario 1; Table 2, rows A–D), all cases were included in the analysis; while when a time-varying confounder existed, when RR = 4, when *M* = 1, and when no censoring occurred (scenario 5; Table 2, row E), more than half of the cases were excluded from the analysis of OR_MH_ and the 95% CI was wider in comparison with OR_G_. Although most cases were used to estimate OR_MH_ when *M* was 10 or more in scenario 5 (Table 2, rows G and H), the estimate of OR_MH_ tended to be unstable in comparison with OR_G_ even when *M* was large, probably because some of the control periods were excluded when OR_MH_ was estimated.

**Table 2:** Odds Ratios and 95% Confidence Intervals Estimated Using the Case–Time-Control Method for Exposure With or Without a Binary Time-Varying Confounder by the Mantel-Haenszel Method and the Weighting Method

		**Mantel-Haenszel Method**							**Weighting Method**	
				**Excluded Cases**		**Included Cases**				
**Scenario **	**No. of Control** **Periods (*M*)**	**OR**_**MH**_ ^**a**^	**95% CI**	**No. of Cases** ^**c**^	**Row %**	**No. of Cases** ^**c**^	**Row %**	**Total No.** **of Cases**	**OR**_**G**_ ^**b**^	**95% CI**
Scenario 1^d^										
A	1	4.13	2.80, 6.10	0	0	177,625	100.0	177,625	4.13	2.80, 6.10
B	2	4.16	2.76, 6.26	0	0	177,625	100.0	177,625	4.16	2.76, 6.26
C	10	4.17	2.69, 6.47	0	0	177,625	100.0	177,625	4.17	2.69, 6.47
D	60	4.18	2.67, 6.54	0	0	177,625	100.0	177,625	4.18	2.67, 6.54
Scenario 5^e^										
E	1	4.20	2.63, 6.70	128,152	53.0	113,616	47.0	241,768	4.18	2.92, 5.99
F	2	4.12	2.65, 6.40	78,240	32.4	163,528	67.6	241,768	4.17	2.85, 6.11
G	10	4.18	2.77, 6.33	5,051	2.1	236,716	97.9	241,768	4.17	2.78, 6.26
H	60	4.35	2.87, 6.59	0	0	241,768	100.0	241,768	4.17	2.75, 6.32

Abbreviations: CI, confidence interval; OR, odds ratio.

^a^ OR estimated by the Mantel-Haenszel method.

^b^ OR estimated by our weighting method (modified Greenland’s likelihood) (8).

^c^ Total number of cases generated by 1,000 iterations.

^d^ Scenario 1 without a binary time-varying confounder where the true rate ratio = 4.0.

^e^ Scenario 5 with a binary time-varying confounder where the true rate ratio = 4.0. Note that the 95% CI is wider and the precision of the OR is lower in the Mantel-Haenszel method than in the weighting method when a time-varying confounder exists in scenario 5.

## DISCUSSION

In our study, we simulated data assuming that a patient was exposed to a medication every day for a period of 60 or more days, analyzing the data using the case-crossover and case–time-control approaches. In the case-crossover study, when no patients were censored after switching to another drug, OR_SCL_ was biased except when *M* = 1 or RR = 1, while OR_MH_ and OR_G_ were unbiased. Those results are compatible with the findings of Vines and Farrington (7) that when pairwise exchangeability holds, OR_MH_ is unbiased, and OR_SCL_ is unbiased when *M* = 1 or RR = 1 even if within-subject exposure dependency exists.

We found that OR_SCL_ is biased due to within-subject dependency when RR = 4 and *M* > 1. This bias cannot be removed by the case–time-control approach and requires the use of the Mantel-Haenszel method or the weighting method.

On the other hand, bias due to censoring occurs in all estimation methods even if RR = 1 or *M* = 1. Bias due to lack of pairwise exchangeability can occur for at least 3 reasons (exposure time trends, persistent users, and censoring). When there are positive exposure time trends, the probability of the (unexposed, exposed) pattern is higher than the probability of the (exposed, unexposed) pattern (13). Persistent users who do not stop taking the drug once they start it have the (unexposed, exposed) pattern only even if a steady state is attained in the population, and persistent user bias may be classified as bias due to lack of pairwise exchangeability which can be removed by the case–time-control method (11). In our simulation, when no patients were censored, pairwise exchangeability held, but when a proportion of patients were censored after switching, the probability of the (unexposed, exposed) pattern was higher than the probability of the (exposed, unexposed) pattern, indicating that pairwise exchangeability did not hold even if a steady state was attained. The results indicated that the case–time-control method removed bias due to censoring. Thus, it is likely that the case–time-control approach removes bias due to lack of pairwise exchangeability in general.

In our simulation study, the time-varying confounder was independent between periods, and estimates of the time-varying confounder in the case-crossover study that were obtained via standard conditional logistic regression (OR_SCL_) were unbiased (OR = 2.0) even when *M* varied. Similarly, ORs for the time-varying confounder of time-controls ($\exp \left(\theta \right)$) were estimated as 1.0 in scenarios 5–8, indicating that pairwise exchangeability held for the confounder. Thus, the behavior of OR_SCL_ for the time-varying confounder in the current study is compatible with our previous findings (8).

Case-crossover studies can suffer from 2 types of biases associated with the analysis method. Bias due to within-subject exposure dependency can be removed with the Mantel-Haenszel method or our weighting method. Bias due to a lack of pairwise exchangeability (from exposure time trends, persistent users, or censoring due to switching) can be removed by using a case–time-control design. When both types of biases exist and RR ≠ 1, use of both the case–time-control design and either the Mantel-Haenszel method or the weighting method is required. Though the use of 1 control period (*M* = 1) can help avoid bias due to within-subject exposure dependency, this usually comes with a price of reduced precision. Unlike our simulation study, in which all patients were exposed and unexposed for at least 60 days even when *M* varied, in real-world data on chronic medication use, some patients may use the drug for a short period only or may restart the drug soon after the exposure ends, and some cases will have the concordant exposure pattern; in these situations, using *M* = 1 will lead to reduced precision. For example, in a case-crossover study on the tricyclic antidepressants–hip fracture association with 1 control period, the OR of 1.18 (95% CI: 0.91, 1.52) was increased to 1.54 (95% CI: 1.28, 1.86) when an earlier control period was used (14, 15).

To test whether within-subject exposure dependency exists in the real-world studies, OR_SCL_ and OR_MH_ can be compared, and if the discrepancy is larger than a prespecified level (e.g., 5% or 10% of OR_MH_), standard conditional logistic regression should not be used (8). The use of a specific length of exposure time window may potentially avoid within-subject exposure dependency and reduce bias from standard conditional logistic regression. The comparison between OR_SCL_ and OR_MH_ may also be useful for selecting the length of the exposure time window that is least biased. However, the primary purpose in choosing the length of one period and any washout periods between them should be to not misclassify the exposure status in each period in terms of the duration of pharmacological effect, rather than to reduce within-subject dependency. In our previous study, we indicated that pairwise exchangeability can be tested by examining whether the proportion of exposed subjects in the population is constant over the study period (8). However, pairwise exchangeability may not hold even when the exposure is stationary over the study period (e.g., due to censoring as in our simulation or persistent users). In these scenarios, a better way to test pairwise exchangeability is to examine whether OR_MH_ is close to 1 in control subjects randomly selected from the population, such as time-controls.

In our simulation, OR_MH_ and OR_G_ for the exposure were similar with or without a time-varying confounder. However, the weighting method may have some advantages when a time-varying confounder exists. For example, OR_G_ was more stable and its 95% CI was narrower compared with OR_MH_, as shown in Table 2.

One limitation of the current study is that the time-varying confounder was assumed to be independent of the exposure and time-varying confounder in different periods. Future studies are warranted to examine how to remove bias when one or more time-varying confounders (e.g., a concomitant drug) have within-subject dependency.

Our study suggests that the requirement of brief exposure as a condition for the use of the case-crossover design may be relaxed and that the case-crossover design may be reliably used for chronic or successive exposure using the Mantel-Haenszel or weighting method, in conjunction with the use of the case–time-control approach when needed.

In conclusion, we have described how the weighting method, with a case–time-control design if needed, can remove biases due to within-subject exposure dependency and lack of pairwise exchangeability caused by switching to another drug or by other factors.

## Supplementary Material

Web_Material_kwad104
