# Supplementary material for: Bias Due to Within-Subject Exposure Dependency With or Without Bias Due to Lack of Pairwise Exchangeability When Exposure Is Chronic in Case-Crossover and Case–Time-Control Studies: A Simulation Study
Source: Am J Epidemiol. 2023 Apr 21;192(10):1701–11. doi: 10.1093/aje/kwad104 (PMC10558192; doi:10.1093/aje/kwad104)
Supplement: Web_Material_kwad104 [file web_material_kwad104.zip › kwad104 Kubota Web Material.pdf]

WEB MATERIAL

Bias Due to Within-Subject Exposure Dependency With or Without Bias Due to Lack of Pairwise Exchangeability When Exposure is Chronic in Case-Crossover and Case–Time-Control Studies: A Simulation Study

Kiyoshi Kubota and Thu-Lan Kelly

Table of Contents Page

Web Appendix: Illustrative examples for understanding concepts in case-crossover and case–time-control studies 2
Web Figure 1: Case-crossover and case–time-control studies 4
Web Figure 2: Case-crossover studies with chronic drug use 5
Web Figure 3: Case-crossover and case–time-control studies when some patients are censored 6
Web Figure 4: Patients (n = 10800-3600Pc) on any day at the steady state 7
Web Figure 5: Illustrative explanation for estimation of weights 8
Web Table 1: List of tables and figures for simulation results for 8 scenarios 9
Web Table 2: Scenario 1 (no time-varying confounder, RR = 4, and no censoring) 10
Web Table 3: Scenario 2 (no time-varying confounder, RR = 4, and 30% censoring) 11
Web Table 4: Scenario 3 (no time-varying confounder, RR = 1, and no censoring) 12
Web Table 5: Scenario 4 (no time-varying confounder, RR = 1, and 30% censoring) 13
Web Figure 6: Scenarios 5 and 6 (with a confounder, RR = 4, and RRz = 2) 14
Web Table 6: Scenario 5 (with a confounder, RR = 4, RRz = 2, and no censoring) 15
Web Table 7: Scenario 6 (with a confounder, RR = 4, RRz = 2, and 30% censoring) 16
Web Figure 7: Scenarios 7 and 8 (with a confounder, RR = 1, and RRz = 2) 17
Web Table 8: Scenario 7 (with a confounder, RR = 1, RRz = 2, and no censoring) 18
Web Table 9: Scenario 8 (with a confounder, RR = 1, RRz = 2, and 30% censoring) 19

Web Appendix

Illustrative Examples for Understanding Concepts in Case-Crossover and Case–Time-Control Studies

To facilitate understanding of concepts in case-crossover and case–time-control studies, we provide 3 examples. The first example illustrates well known bias due to exposure time-trends which can be removed by the case–time-control approach. The second one illustrates bias due to within-subject exposure dependency associated with chronic drug use. The third one illustrates bias due to censoring which may occur even if there is no exposure time trend and can be removed by the case–time-control approach as for the first example.

Bias due to exposure time trends in case-crossover studies and case–time-control approach to remove the bias (see Web Figure 1)

Assume that during the study duration with two periods (a control period followed by a case period), the size of the population with a potential indication of a drug is 900,000 and does not change. We consider two scenarios (a) without time trend where the proportion of those exposed to the drug (P_{ex}, in Web Figure 1) is 1/3 and does not change, and (b) with time trend where P_{ex} increases from 1/3 to 2/3 (e.g., the drug is a newly marketed drug). The occurrence of an event of interest is 1 and 4 in 10,000 per period when unexposed and exposed respectively (i.e., rate ratio (RR) = 4).

Assume that the use of the drug in the case period is independent from that in the control period. In scenario (a), the expected number of discordant cases exposed (n_1) and unexposed (n_0) at the case period will be 80 and 20, respectively and odds ratio in the case-crossover study (OR_{case}) is 4.0 and unbiased. In scenario (b), n_1 = 160 and n_0 = 10, and OR_{case} is over-estimated as 16.0. With OR of 170 discordant time-controls (OR_{tc}) = 4, OR in the case–time-control study (OR_{case} / OR_{tc}) is 4 and unbiased.

Bias due to within-subject exposure dependency with chronic drug use in case-crossover studies (see Web Figure 2)

Assume that a case-crossover study with one case period and two control periods is conducted. In scenario (a), exposure occurs independently between periods (exposure probability = 0.5) and in scenario (b), exposure is chronic and once a patient becomes exposed or unexposed, the same exposure status continues for at least 3 successive periods. The occurrence of an outcome of interest is 1 and 4 in 10,000 per period when unexposed and exposed, respectively (i.e., rate ratio (RR) = 4).

In scenario (a) where 8 exposure patterns occur with the same probability of which 6 are discordant, the odds ratio (OR) estimated by the standard conditional logistic regression (OR_{SCL}) and that by the Mantel-Haenszel (OR_{MH}) are unbiased. In scenario (b), only 6 exposure patterns occur of which 4 are discordant. An unbiased OR_{MH} is

estimated but OR_{SCL} is biased. Bias in scenario (b) cannot be removed by the case–time-control approach because OR of time-controls (OR_{tc}) = 1 both in scenarios (a) and (b).

Bias due to censoring in a case-crossover and case–time-control studies with one control period (see Web Figure 3)

Assume that a case-crossover study with one case period and one control period is conducted in a dynamic population where patients enter and leave the cohort at every period (Web Figure 3). Assume that 200,000 patients per period start a drug after a period of non-use and enter the cohort. They use the drug for only 1 period. A half of patients stop the drug and become unexposed and remain in the cohort as unexposed patients, but another half of patients switch to another drug and censored.

When the exposure pattern becomes stationary (or the "steady-state" is attained), the proportion of those exposed (2/3) does not change over time and in each period, the cohort includes 300,000 (200,000 exposed and 100,000 unexposed) patients. In a case-crossover study conducted at period 1 (and at any period when the exposure pattern is stationary), the odds ratio (OR) is overestimated as 8. This bias can be removed by the case–time-control approach because OR of time-controls (OR_{tc}) is 2.0. This example indicates that bias due to censoring which can be removed by the case–time-control approach may take place even if there is no exposure time trend. In the current study, the term "bias due to lack of pairwise exchangeability" is used which includes both bias due to exposure time trend and bias due to censoring.

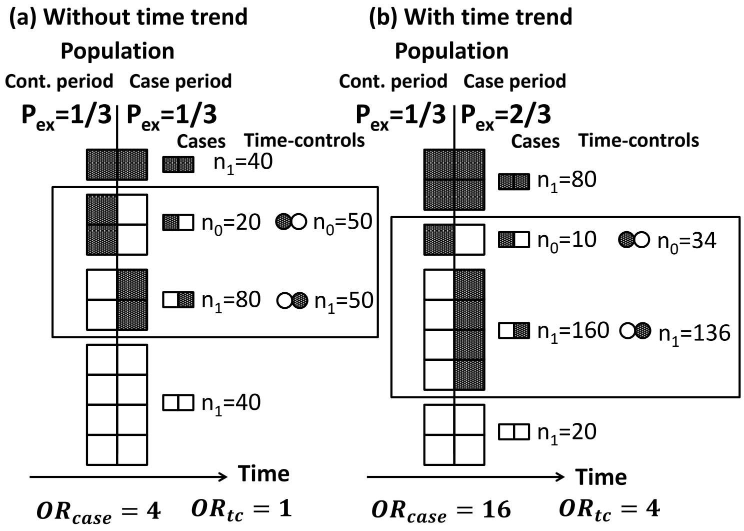

Web Figure 1. Case-crossover and case–time-control studies

One large square denotes 100,000 patients exposed (filled) and those unexposed (open) in the population. Small squares and circles denote cases and time-controls, respectively. Area surrounded by the framed rectangle includes discordant cases and time-controls. OR in the case–time-control study is OR_{case}/OR_{tc}.

Cont. period: control period; n_1: expected number of exposed cases and time-controls; n_0: expected number of unexposed cases and time-controls; P_{ex}: proportion of exposed in the population. OR_{case}: odds ratio of cases in case-crossover study. OR_{tc}: odds ratio of time-controls; n_1: expected number of exposed cases or time-controls; n_0: expected number of unexposed cases or time-controls.

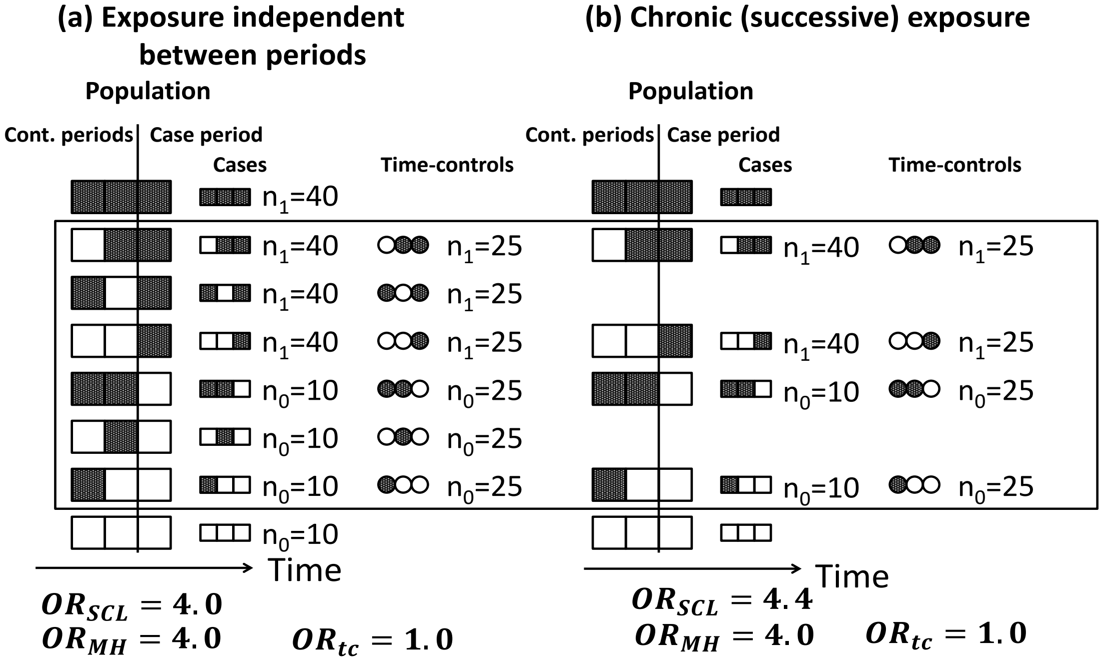

Web Figure 2. Case-crossover studies with chronic drug use

One large square denotes 100,000 patients exposed (filled) and unexposed (open) in the population except for concordant pattern (always exposed (top) or unexposed (bottom)) in scenario (b), which may be larger than 100,000 when exposed or unexposed period lasts for more than 3 periods. Small squares and circles denote cases and time-controls, respectively.

Cont. periods: control periods; n_1: expected number of exposed cases and time-controls; n_0: expected number of unexposed cases and time-controls; OR_{SCL}: odds ratio (OR) by the standard conditional logistic regression; OR_{MH}: OR by the Mantel-Haenszel method; OR_{tc}: OR of time-controls.

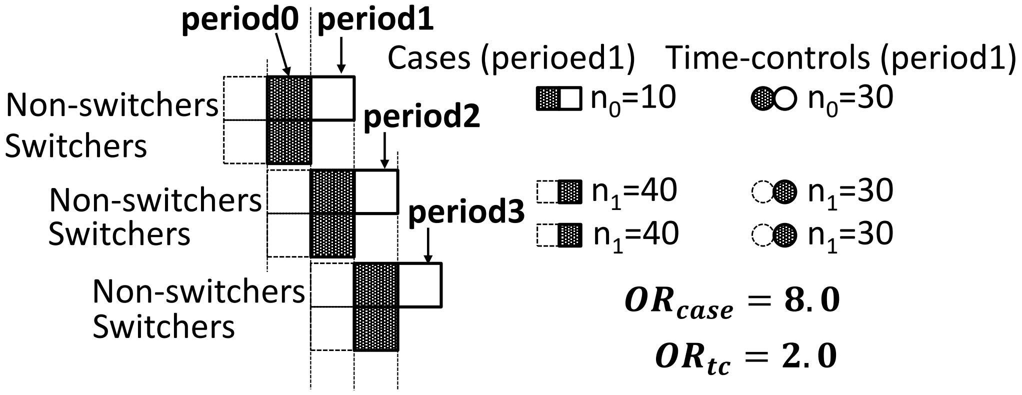

Web Figure 3. Case-crossover and case–time-control studies when some patients are censored

One large square denotes 100,000 patients exposed (filled) and unexposed (open) in the dynamic population where 200,000 patients per period start the drug and enter the cohort after a period of non-use. Small squares and circles denote cases and time-controls, respectively. Patients use the drug for only 1 period. A half of them stop the drug and remain in the cohort as unexposed patients while the other half switch to another drug and censored.

n_1: expected number of exposed cases and time-controls; n_0: expected number of unexposed cases and time-controls; OR_{case}: odds ratio (OR) of cases in the case-crossover study; OR_{tc}: OR of time-controls.

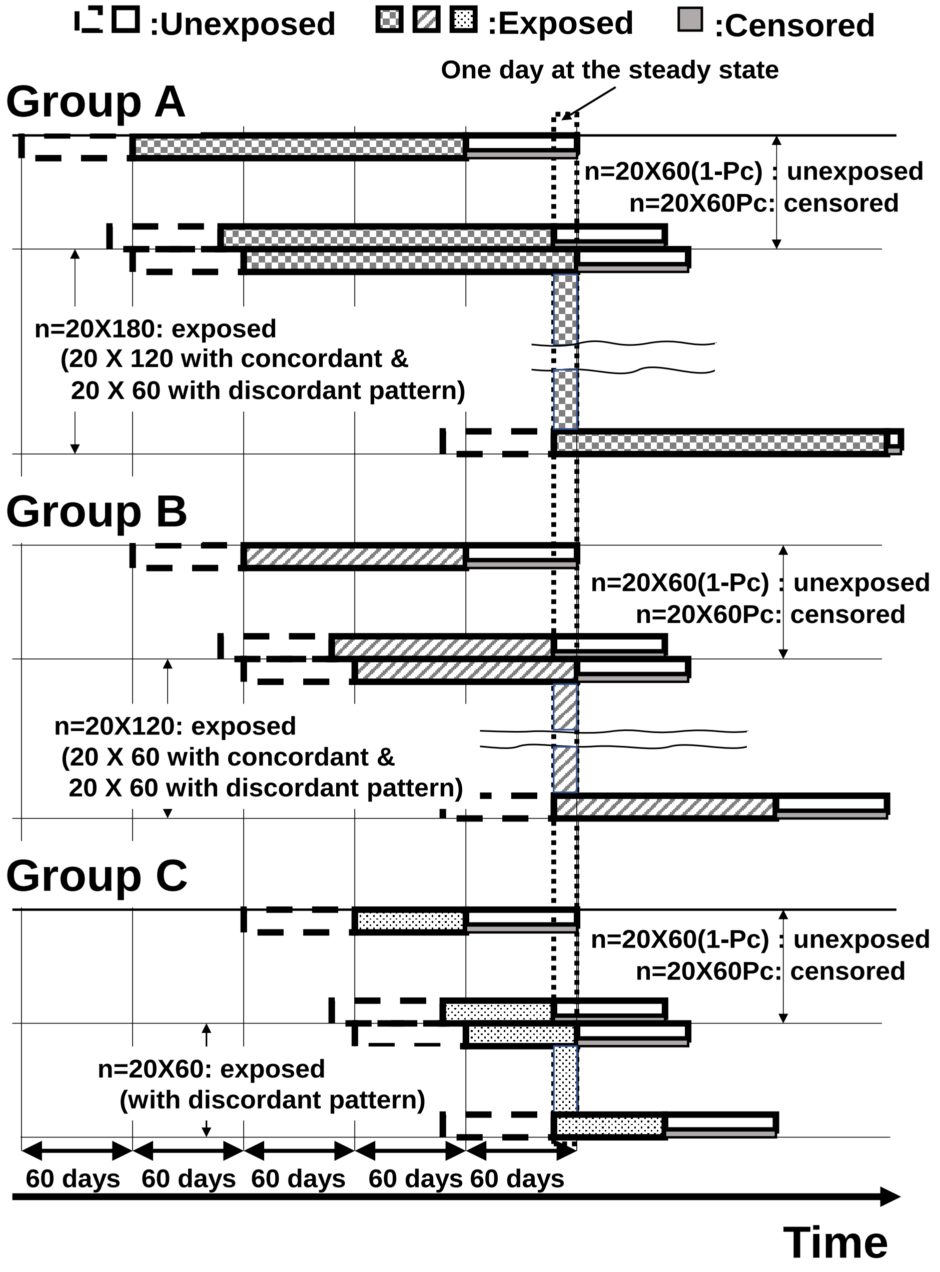

Web Figure 4. Patients (n = 10800-3600Pc) at any day at the steady state

(10800-3600Pc) patients at the steady state correspond to (10800-3600Pc) days in Figure 1A in the text.

Cases

		PT_{10i}	PT_{01i}	$n_1 * PT_{10i}$	$n_0 * PT_{01i}$	m_i^1	m_i^0	π_{10}/m_i^1	$1/m_i^0$
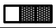	n_1=40	1	—	40	—	2	1	0.5	1
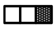	n_1=40	2	—	80	—	1	2	1	0.5
	a_1=80								
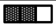	n_0=10	—	2	—	20	2	1	0.5	1
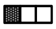	n_0=10	—	1	—	10	1	2	1	0.5
	a_0=20								

Time-controls

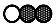	n_1=25	1	—	25	—	2	1	0.5	1
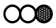	n_1=25	2	—	50	—	1	2	1	0.5
	b_1=50								
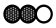	n_0=25	—	2	—	50	2	1	0.5	1
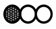	n_0=25	—	1	—	25	1	2	1	0.5
	b_0=50								

	ΣPT_{10i}			195					
	ΣPT_{01i}				105				

$$\pi_{10} = \frac{\sum_i PT_{01i}/(a_0 + b_0)}{\sum_i PT_{10i}/(a_1 + b_1)} = \frac{105/(20+50)}{195/(80+50)} = 1.0$$

Web Figure 5. Illustrative explanation for estimation of weights

Procedures to estimate weights (π_{10}/m_i^1 for exposed periods and $1/m_i^0$ for unexposed periods) is illustrated using 100 cases and 100 time-controls in scenario (b) of Web Figure 2 as an example.

n_1: number of exposed cases and time-controls (130); n_0: number of unexposed cases and time-controls (70); a_1: number of exposed cases (80); a_0: number of unexposed cases (20); b_1: number of exposed time-controls (50); b_0: number of unexposed time-controls (50); PT_{10i}: number of unexposed control periods of subject i with an exposed case period; PT_{01i}: number of exposed control periods of subject i with an unexposed case period; m_i^1: number of exposed (case and control) periods of subject i; m_i^0: number of unexposed (case and control) periods of subject i.

Web Table 1. List of tables and figures for simulation results for 8 scenarios

Scenario	RR	RRz	Pc [a]	Figure	Table
Scenario 1	4.0	-	0%	A) B) Figure 2	Web Table 2
Scenario 2	4.0	-	30%	C) D) Figure 2	Web Table 3
Scenario 3	1.0	-	0%	A) B) Figure 3	Web Table 4
Scenario 4	1.0	-	30%	C) D) Figure 3	Web Table 5
Scenario 5	4.0	2.0	0%	A) B) C) Web Figure 6	Web Table 6
Scenario 6	4.0	2.0	30%	D) E) F) Web Figure 6	Web Table 7
Scenario 7	1.0	2.0	0%	A) B) C) Web Figure 7	Web Table 8
Scenario 8	1.0	2.0	30%	D) E) F) Web Figure 7	Web Table 9

Figures and tables for simulation results obtained by 1000 iterations for 8 scenarios are shown.

[a] Proportion of those who switch to another drug and are censored when the exposure period ends.

RR: rate ratio for exposure; RRz: rate ratio for a binary time-varying confounder (z).

Web Table 2. Odds Ratios (ORs) for Scenario 1 (no time-varying confounder, RR = 4, and no censoring)

	CXO study, cases			CXO study, time-controls			Case-time-control study		
M	OR_{SCL}	OR_{MH}	OR_G	OR_{SCL}	OR_{MH}	OR_G	OR_{SCL}	OR_{MH}	OR_G
**1**	4.1 (2.8-6.1)	4.1 (2.8-6.1)	4.1 (2.8-6.1)	1.0 (0.8-1.4)	1.0 (0.8-1.4)	1.0 (0.8-1.4)	4.0 (2.5-6.5)	4.0 (2.5-6.5)	4.0 (2.5-6.5)
**2**	4.8 (3.1-7.3)	4.2 (2.8-6.3)	4.2 (2.8-6.3)	1.0 (0.7-1.5)	1.0 (0.8-1.4)	1.0 (0.8-1.4)	4.6 (2.7-8.1)	4.0 (2.4-6.8)	4.0 (2.5-6.5)
**3**	5.2 (3.3-8.2)	4.2 (2.7-6.3)	4.2 (2.7-6.3)	1.0 (0.7-1.5)	1.0 (0.8-1.4)	1.0 (0.8-1.4)	5.1 (2.8-9.2)	4.0 (2.4-6.9)	4.0 (2.5-6.5)
**4**	5.6 (3.5-8.9)	4.2 (2.7-6.4)	4.2 (2.7-6.4)	1.0 (0.7-1.5)	1.0 (0.7-1.4)	1.0 (0.7-1.4)	5.4 (2.9-10.0)	4.0 (2.4-6.9)	4.0 (2.5-6.5)
**5**	5.9 (3.6-9.5)	4.2 (2.7-6.4)	4.2 (2.7-6.4)	1.0 (0.7-1.6)	1.0 (0.7-1.4)	1.0 (0.7-1.4)	5.6 (3.0-10.7)	4.0 (2.4-6.9)	4.0 (2.5-6.5)
**6**	6.1 (3.7-10.0)	4.2 (2.7-6.4)	4.2 (2.7-6.4)	1.0 (0.7-1.6)	1.0 (0.7-1.4)	1.0 (0.7-1.4)	5.8 (3.0-11.3)	4.1 (2.3-7.0)	4.0 (2.5-6.5)
**10**	6.6 (3.9-11.3)	4.2 (2.7-6.5)	4.2 (2.7-6.5)	1.0 (0.7-1.6)	1.0 (0.7-1.4)	1.0 (0.7-1.4)	6.4 (3.2-12.9)	4.1 (2.3-7.1)	4.0 (2.5-6.5)
**12**	6.8 (4.0-11.7)	4.2 (2.7-6.5)	4.2 (2.7-6.5)	1.0 (0.7-1.6)	1.0 (0.7-1.4)	1.0 (0.7-1.4)	6.5 (3.2-13.4)	4.1 (2.3-7.1)	4.0 (2.5-6.5)
**15**	7.0 (4.0-12.2)	4.2 (2.7-6.5)	4.2 (2.7-6.5)	1.0 (0.7-1.7)	1.0 (0.7-1.4)	1.0 (0.7-1.4)	6.7 (3.2-14.1)	4.1 (2.3-7.1)	4.0 (2.5-6.5)
**20**	7.3 (4.1-12.8)	4.2 (2.7-6.5)	4.2 (2.7-6.5)	1.0 (0.6-1.7)	1.0 (0.7-1.4)	1.0 (0.7-1.4)	7.0 (3.3-14.7)	4.1 (2.3-7.1)	4.0 (2.5-6.5)
**30**	7.5 (4.2-13.5)	4.2 (2.7-6.5)	4.2 (2.7-6.5)	1.0 (0.6-1.7)	1.0 (0.7-1.4)	1.0 (0.7-1.4)	7.2 (3.3-15.6)	4.1 (2.3-7.1)	4.0 (2.5-6.5)
**60**	7.8 (4.3-14.4)	4.2 (2.7-6.5)	4.2 (2.7-6.5)	1.0 (0.6-1.7)	1.0 (0.7-1.4)	1.0 (0.7-1.4)	7.5 (3.4-16.6)	4.1 (2.3-7.2)	4.0 (2.5-6.5)

Odds ratios (ORs) by the standard conditional logistic regression (OR_{SCL}), by the Mantel-Haenszel method (OR_{MH}), by the weighting method (OR_G) and their 95% confidence intervals estimated in case-crossover (CXO) studies for cases and time-controls and in case–time-control studies for Scenario 1. Data in this table is also presented in Figure 2A (CXO study, cases) and B (case–time-control study) in the text for selected values of M.

Web Table 3. Odds Ratios (ORs) for Scenario 2 (no time-varying confounder, RR = 4, and 30% censoring)

	CXO study, cases			CXO study, time-controls			Case-time-control study		
M	OR_{SCL}	OR_{MH}	OR_G	OR_{SCL}	OR_{MH}	OR_G	OR_{SCL}	OR_{MH}	OR_G
**1**	5.9 (3.8-9.3)	5.9 (3.8-9.3)	5.9 (3.8-9.3)	1.5 (1.1-2.0)	1.5 (1.1-2.0)	1.5 (1.1-2.0)	4.0 (2.3-7.0)	4.0 (2.3-7.0)	4.0 (2.3-7.0)
**2**	7.0 (4.3-11.4)	5.9 (3.7-9.5)	5.9 (3.7-9.5)	1.5 (1.1-2.2)	1.5 (1.1-2.0)	1.5 (1.1-2.0)	4.5 (2.4-8.4)	4.0 (2.2-7.2)	4.0 (2.3-7.0)
**3**	7.8 (4.7-12.9)	5.9 (3.6-9.7)	5.9 (3.6-9.7)	1.6 (1.1-2.4)	1.5 (1.1-2.1)	1.5 (1.1-2.1)	4.9 (2.5-9.4)	4.0 (2.2-7.4)	4.0 (2.3-7.0)
**4**	8.4 (4.9-14.2)	5.9 (3.6-9.8)	5.9 (3.6-9.8)	1.6 (1.1-2.5)	1.5 (1.1-2.1)	1.5 (1.1-2.1)	5.1 (2.6-10.2)	4.0 (2.2-7.4)	4.0 (2.3-7.0)
**5**	8.8 (5.1-15.2)	5.9 (3.6-9.8)	5.9 (3.6-9.8)	1.7 (1.1-2.5)	1.5 (1.1-2.1)	1.5 (1.1-2.1)	5.3 (2.6-10.8)	4.0 (2.2-7.4)	4.0 (2.3-7.0)
**6**	9.2 (5.3-16.0)	5.9 (3.6-9.8)	5.9 (3.6-9.8)	1.7 (1.1-2.6)	1.5 (1.1-2.1)	1.5 (1.1-2.1)	5.5 (2.7-11.3)	4.0 (2.2-7.5)	4.0 (2.3-7.0)
**10**	10.3 (5.7-18.5)	5.9 (3.6-9.9)	5.9 (3.6-9.9)	1.7 (1.1-2.7)	1.5 (1.0-2.1)	1.5 (1.0-2.1)	6.0 (2.8-12.9)	4.0 (2.1-7.5)	4.0 (2.3-7.0)
**12**	10.6 (5.8-19.3)	5.9 (3.6-9.9)	5.9 (3.6-9.9)	1.7 (1.1-2.8)	1.5 (1.0-2.1)	1.5 (1.0-2.1)	6.2 (2.8-13.4)	4.0 (2.1-7.5)	4.0 (2.3-7.0)
**15**	11.0 (6.0-20.3)	5.9 (3.5-9.9)	5.9 (3.5-9.9)	1.7 (1.1-2.8)	1.5 (1.0-2.1)	1.5 (1.0-2.1)	6.3 (2.9-14.1)	4.0 (2.1-7.6)	4.0 (2.3-7.0)
**20**	11.5 (6.1-21.5)	5.9 (3.5-10.0)	5.9 (3.5-10.0)	1.8 (1.1-2.9)	1.5 (1.0-2.1)	1.5 (1.0-2.1)	6.6 (2.9-14.8)	4.0 (2.1-7.6)	4.0 (2.3-7.0)
**30**	12.1 (6.3-23.2)	5.9 (3.5-10.0)	5.9 (3.5-10.0)	1.8 (1.1-2.9)	1.5 (1.0-2.1)	1.5 (1.0-2.1)	6.8 (2.9-15.9)	4.0 (2.1-7.6)	4.0 (2.3-7.0)
**60**	12.7 (6.5-25.2)	5.9 (3.5-10.0)	5.9 (3.5-10.0)	1.8 (1.1-3.0)	1.5 (1.0-2.1)	1.5 (1.0-2.1)	7.1 (3.0-17.1)	4.0 (2.1-7.6)	4.0 (2.3-7.0)

Odds ratios (ORs) by the standard conditional logistic regression (OR_{SCL}), by the Mantel-Haenszel method (OR_{MH}), by the weighting method (OR_G) and their 95% confidence intervals estimated in case-crossover (CXO) studies for cases and time-controls and in case–time-control studies for Scenario 2. Data in this table is also presented in Figure 2C (CXO study, cases) and D (case–time-control study) in the text for selected values of M.

Web Table 4. Odds Ratios (ORs) for Scenario 3 (no time-varying confounder, RR = 1, and no censoring)

	**CXO study, cases**			**CXO study, time-controls**			**Case-time-control study**		
M	**OR_{SCL}**	**OR_{MH}**	**OR_G**	**OR_{SCL}**	**OR_{MH}**	**OR_G**	**OR_{SCL}**	**OR_{MH}**	**OR_G**
**1**	1.0 (0.6-1.6)	1.0 (0.6-1.6)	1.0 (0.6-1.6)	1.0 (0.6-1.6)	1.0 (0.6-1.6)	1.0 (0.6-1.6)	1.0 (0.5-1.9)	1.0 (0.5-1.9)	1.0 (0.5-1.9)
**2**	1.0 (0.6-1.8)	1.0 (0.6-1.7)	1.0 (0.6-1.7)	1.0 (0.6-1.8)	1.0 (0.6-1.7)	1.0 (0.6-1.7)	1.0 (0.5-2.2)	1.0 (0.5-2.0)	1.0 (0.5-1.9)
**3**	1.0 (0.6-1.9)	1.0 (0.6-1.7)	1.0 (0.6-1.7)	1.0 (0.6-1.8)	1.0 (0.6-1.7)	1.0 (0.6-1.7)	1.0 (0.4-2.3)	1.0 (0.5-2.0)	1.0 (0.5-1.9)
**4**	1.0 (0.5-2.0)	1.0 (0.6-1.7)	1.0 (0.6-1.7)	1.0 (0.5-1.9)	1.0 (0.6-1.7)	1.0 (0.6-1.7)	1.0 (0.4-2.5)	1.0 (0.5-2.1)	1.0 (0.5-1.9)
**5**	1.0 (0.5-2.0)	1.0 (0.6-1.7)	1.0 (0.6-1.7)	1.0 (0.5-2.0)	1.0 (0.6-1.7)	1.0 (0.6-1.7)	1.0 (0.4-2.6)	1.0 (0.5-2.1)	1.0 (0.5-1.9)
**6**	1.0 (0.5-2.0)	1.0 (0.6-1.7)	1.0 (0.6-1.7)	1.0 (0.5-2.0)	1.0 (0.6-1.7)	1.0 (0.6-1.7)	1.0 (0.4-2.6)	1.0 (0.5-2.1)	1.0 (0.5-1.9)
**10**	1.0 (0.5-2.1)	1.0 (0.6-1.7)	1.0 (0.6-1.7)	1.0 (0.5-2.1)	1.0 (0.6-1.7)	1.0 (0.6-1.7)	1.0 (0.4-2.8)	1.0 (0.5-2.1)	1.0 (0.5-1.9)
**12**	1.0 (0.5-2.2)	1.0 (0.6-1.8)	1.0 (0.6-1.8)	1.0 (0.5-2.1)	1.0 (0.6-1.7)	1.0 (0.6-1.7)	1.0 (0.4-2.9)	1.0 (0.5-2.1)	1.0 (0.5-1.9)
**15**	1.0 (0.5-2.2)	1.0 (0.6-1.8)	1.0 (0.6-1.8)	1.0 (0.5-2.1)	1.0 (0.6-1.7)	1.0 (0.6-1.7)	1.0 (0.3-2.9)	1.0 (0.5-2.1)	1.0 (0.5-1.9)
**20**	1.0 (0.5-2.2)	1.0 (0.6-1.8)	1.0 (0.6-1.8)	1.0 (0.5-2.2)	1.0 (0.6-1.7)	1.0 (0.6-1.7)	1.0 (0.3-3.0)	1.0 (0.5-2.2)	1.0 (0.5-1.9)
**30**	1.0 (0.5-2.3)	1.0 (0.6-1.8)	1.0 (0.6-1.8)	1.0 (0.5-2.2)	1.0 (0.6-1.7)	1.0 (0.6-1.7)	1.0 (0.3-3.0)	1.0 (0.5-2.2)	1.0 (0.5-1.9)
**60**	1.0 (0.5-2.3)	1.0 (0.6-1.8)	1.0 (0.6-1.8)	1.0 (0.5-2.2)	1.0 (0.6-1.7)	1.0 (0.6-1.7)	1.0 (0.3-3.1)	1.0 (0.5-2.2)	1.0 (0.5-1.9)

Odds ratios (ORs) by the standard conditional logistic regression (OR_{SCL}), by the Mantel-Haenszel method (OR_{MH}), by the weighting method (OR_G) and their 95% confidence intervals estimated in case-crossover (CXO) studies for cases and time-controls and in case–time-control studies for Scenario 3. Data in this table is also presented in Figure 3A (CXO study, cases) and B (case–time-control study) in the text for selected values of M.

Web Table 5. Odds Ratios (ORs) for Scenario 4 (no time-varying confounder, RR = 1, and 30% censoring)

	CXO study, cases			CXO study, time-controls			Case-time-control study		
M	OR_{SCL}	OR_{MH}	OR_G	OR_{SCL}	OR_{MH}	OR_G	OR_{SCL}	OR_{MH}	OR_G
**1**	1.4 (0.9-2.4)	1.4 (0.9-2.4)	1.4 (0.9-2.4)	1.5 (0.9-2.5)	1.5 (0.9-2.5)	1.5 (0.9-2.5)	1.0 (0.5-2.1)	1.0 (0.5-2.1)	1.0 (0.5-2.1)
**2**	1.5 (0.8-2.8)	1.4 (0.8-2.5)	1.4 (0.8-2.5)	1.5 (0.8-2.9)	1.5 (0.8-2.6)	1.5 (0.8-2.6)	1.0 (0.4-2.4)	1.0 (0.4-2.2)	1.0 (0.5-2.1)
**3**	1.5 (0.8-3.0)	1.4 (0.8-2.5)	1.4 (0.8-2.5)	1.6 (0.8-3.1)	1.5 (0.8-2.6)	1.5 (0.8-2.6)	1.0 (0.4-2.5)	1.0 (0.4-2.2)	1.0 (0.5-2.1)
**4**	1.6 (0.8-3.2)	1.4 (0.8-2.6)	1.4 (0.8-2.6)	1.6 (0.8-3.3)	1.5 (0.8-2.6)	1.5 (0.8-2.6)	1.0 (0.4-2.7)	1.0 (0.4-2.2)	1.0 (0.5-2.1)
**5**	1.6 (0.8-3.3)	1.4 (0.8-2.6)	1.4 (0.8-2.6)	1.6 (0.8-3.4)	1.5 (0.8-2.6)	1.5 (0.8-2.6)	1.0 (0.3-2.8)	1.0 (0.4-2.3)	1.0 (0.5-2.1)
**6**	1.6 (0.8-3.4)	1.4 (0.8-2.6)	1.4 (0.8-2.6)	1.7 (0.8-3.5)	1.5 (0.8-2.6)	1.5 (0.8-2.6)	1.0 (0.3-2.8)	1.0 (0.4-2.2)	1.0 (0.5-2.1)
**10**	1.6 (0.7-3.6)	1.4 (0.8-2.6)	1.4 (0.8-2.6)	1.7 (0.8-3.8)	1.5 (0.8-2.7)	1.5 (0.8-2.7)	1.0 (0.3-3.0)	1.0 (0.4-2.3)	1.0 (0.5-2.1)
**12**	1.7 (0.7-3.7)	1.4 (0.8-2.6)	1.4 (0.8-2.6)	1.7 (0.8-3.8)	1.5 (0.8-2.7)	1.5 (0.8-2.7)	1.0 (0.3-3.1)	1.0 (0.4-2.3)	1.0 (0.5-2.1)
**15**	1.7 (0.7-3.8)	1.4 (0.8-2.6)	1.4 (0.8-2.6)	1.7 (0.8-3.9)	1.5 (0.8-2.7)	1.5 (0.8-2.7)	1.0 (0.3-3.2)	1.0 (0.4-2.3)	1.0 (0.5-2.1)
**20**	1.7 (0.7-3.9)	1.4 (0.8-2.6)	1.4 (0.8-2.6)	1.7 (0.7-4.0)	1.5 (0.8-2.7)	1.5 (0.8-2.7)	1.0 (0.3-3.2)	1.0 (0.4-2.3)	1.0 (0.5-2.1)
**30**	1.7 (0.7-4.0)	1.4 (0.8-2.6)	1.4 (0.8-2.6)	1.7 (0.7-4.1)	1.5 (0.8-2.7)	1.5 (0.8-2.7)	1.0 (0.3-3.3)	1.0 (0.4-2.3)	1.0 (0.5-2.1)
**60**	1.7 (0.7-4.1)	1.4 (0.8-2.6)	1.4 (0.8-2.6)	1.8 (0.7-4.2)	1.5 (0.8-2.7)	1.5 (0.8-2.7)	1.0 (0.3-3.4)	1.0 (0.4-2.3)	1.0 (0.5-2.1)

Odds ratios (ORs) by the standard conditional logistic regression (OR_{SCL}), by the Mantel-Haenszel method (OR_{MH}), by the weighting method (OR_G) and their 95% confidence intervals estimated in case-crossover (CXO) studies for cases and time-controls and in case–time-control studies for Scenario 4. Data in this table is also presented in Figure 3C (CXO study, cases) and D (case–time-control study) in the text for selected values of M.

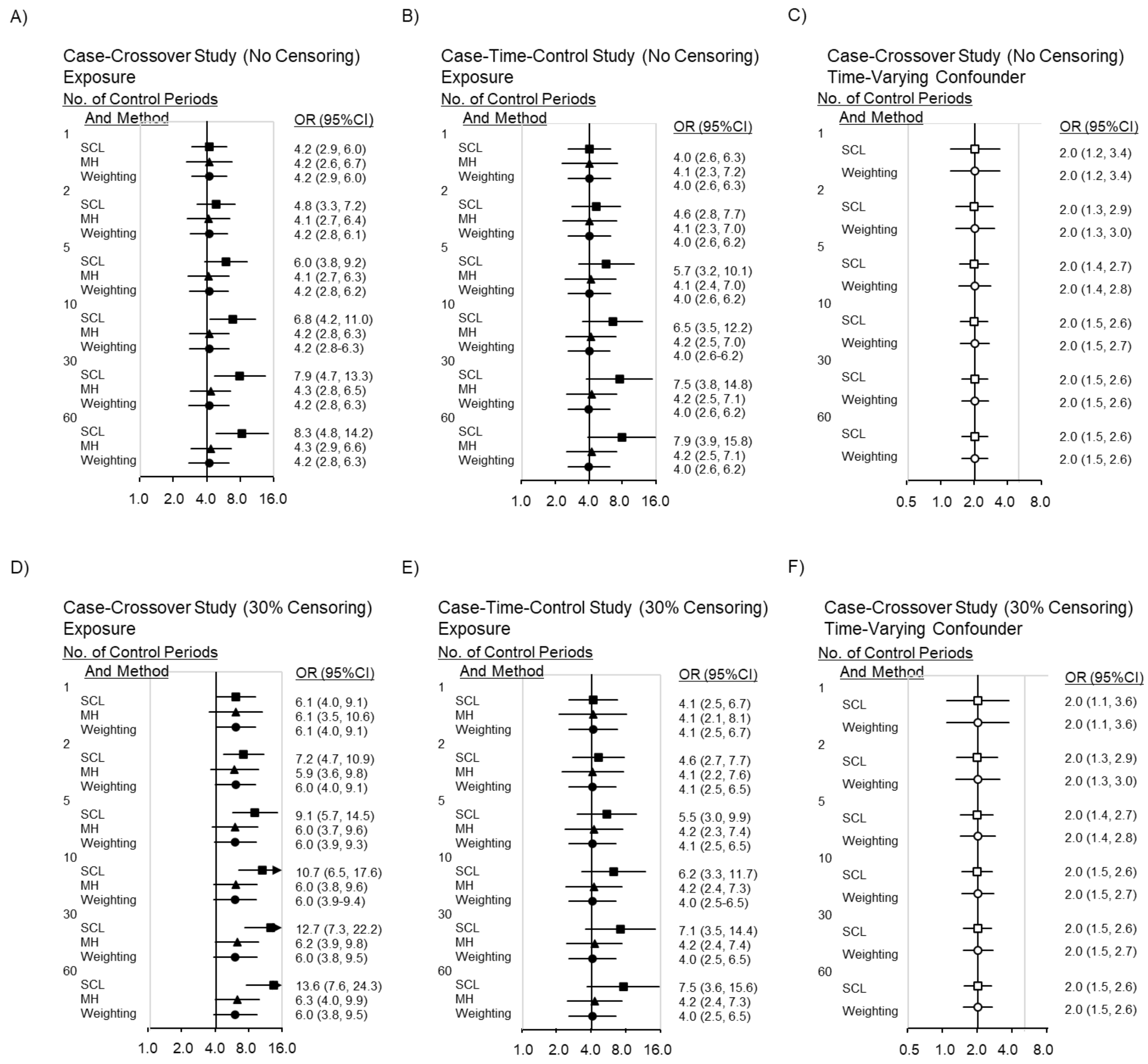

Web Figure 6. Odds ratios (ORs) with 95% CIs estimated in case-crossover studies for exposure and binary time-varying confounder in Scenarios 5 and 6 (RR = 4, RRz = 2)

Web Figures 6A, 6B, and 6C show ORs for exposure in case-crossover studies, exposure in case–time-control studies, and time-varying confounder of cases in case-crossover studies, respectively for Scenario 5 where no patients are censored at the end of the exposed period. Web Figures 6D, 6E, and 6F show ORs for exposure in case-crossover studies, exposure in case–time-control studies, and time-varying confounder of cases in case-crossover study, respectively for Scenario 6 where 30% of patients are censored at the end of the exposed period.

RR: true rate ratio for exposure; RRz: true rate ratio for time-varying confounder; OR: estimated odds ratio; SCL: OR by the standard conditional logistic regression; MH: OR by the Mantel-Haenszel method; Weighting: OR by the weighting method; CI: confidence interval.

Web Table 6. Odds Ratios (ORs) for Scenarios 5 (with a binary time-varying confounder, RR = 4, and no censoring)

	**Case-crossover study, cases**					**Case-crossover study, time-controls**					**Case-time-control study**				
	**Exposure**			**Time-varying confounder**		**Exposure**			**Time-varying confounder**		**Exposure**			**Time-varying confounder**	
**M**	**OR_{SCL}**	**OR_{MH}**	**OR_G**	**OR_{SCL}**	**OR_G**	**OR_{SCL}**	**OR_{MH}**	**OR_G**	**OR_{SCL}**	**OR_G**	**OR_{SCL}**	**OR_{MH}**	**OR_G**	**OR_{SCL}**	**OR_G**
**1**	4.2 (2.9-6.0)	4.2 (2.6-6.7)	4.2 (2.9-6.0)	2.0 (1.2-3.4)	2.0 (1.2-3.4)	1.0 (0.8-1.4)	1.0 (0.7-1.4)	1.0 (0.8-1.4)	1.0 (0.7-1.5)	1.0 (0.7-1.5)	4.0 (2.6-6.3)	4.1 (2.3-7.2)	4.0 (2.6-6.3)	2.0 (1.0-3.9)	2.0 (1.0-3.9)
**2**	4.8 (3.3-7.2)	4.1 (2.7-6.4)	4.2 (2.8-6.1)	2.0 (1.3-2.9)	2.0 (1.3-3.0)	1.0 (0.8-1.4)	1.0 (0.7-1.4)	1.0 (0.8-1.4)	1.0 (0.7-1.4)	1.0 (0.7-1.4)	4.6 (2.8-7.7)	4.1 (2.3-7.0)	4.0 (2.6-6.2)	2.0 (1.2-3.4)	2.0 (1.2-3.5)
**3**	5.3 (3.5-8.0)	4.1 (2.7-6.3)	4.2 (2.8-6.2)	2.0 (1.4-2.8)	2.0 (1.4-2.9)	1.0 (0.7-1.5)	1.0 (0.7-1.4)	1.0 (0.8-1.4)	1.0 (0.7-1.4)	1.0 (0.7-1.4)	5.1 (3.0-8.7)	4.1 (2.4-7.0)	4.0 (2.6-6.2)	2.0 (1.2-3.2)	2.0 (1.2-3.3)
**4**	5.7 (3.7-8.7)	4.1 (2.7-6.3)	4.2 (2.8-6.2)	2.0 (1.4-2.7)	2.0 (1.4-2.8)	1.0 (0.7-1.5)	1.0 (0.7-1.4)	1.0 (0.8-1.4)	1.0 (0.7-1.4)	1.0 (0.7-1.4)	5.4 (3.1-9.5)	4.1 (2.4-7.0)	4.0 (2.6-6.2)	2.0 (1.3-3.2)	2.0 (1.2-3.3)
**5**	6.0 (3.8-9.2)	4.1 (2.7-6.3)	4.2 (2.8-6.2)	2.0 (1.4-2.7)	2.0 (1.4-2.8)	1.0 (0.7-1.5)	1.0 (0.7-1.4)	1.0 (0.8-1.4)	1.0 (0.7-1.4)	1.0 (0.7-1.4)	5.7 (3.2-10.1)	4.1 (2.4-7.0)	4.0 (2.6-6.2)	2.0 (1.3-3.1)	2.0 (1.3-3.2)
**6**	6.2 (3.9-9.7)	4.1 (2.7-6.3)	4.2 (2.8-6.2)	2.0 (1.5-2.7)	2.0 (1.5-2.8)	1.0 (0.7-1.5)	1.0 (0.7-1.4)	1.0 (0.8-1.4)	1.0 (0.7-1.3)	1.0 (0.7-1.4)	5.9 (3.3-10.7)	4.1 (2.4-6.9)	4.0 (2.6-6.2)	2.0 (1.3-3.1)	2.0 (1.3-3.2)
**10**	6.8 (4.2-11.0)	4.2 (2.8-6.3)	4.2 (2.8-6.3)	2.0 (1.5-2.6)	2.0 (1.5-2.7)	1.0 (0.7-1.6)	1.0 (0.7-1.4)	1.0 (0.8-1.4)	1.0 (0.7-1.3)	1.0 (0.7-1.4)	6.5 (3.5-12.2)	4.2 (2.5-7.0)	4.0 (2.6-6.2)	2.0 (1.3-3.0)	2.0 (1.3-3.1)
**12**	7.0 (4.3-11.5)	4.2 (2.8-6.4)	4.2 (2.8-6.3)	2.0 (1.5-2.6)	2.0 (1.5-2.7)	1.0 (0.7-1.6)	1.0 (0.7-1.4)	1.0 (0.8-1.4)	1.0 (0.7-1.3)	1.0 (0.7-1.3)	6.7 (3.5-12.7)	4.2 (2.5-7.0)	4.0 (2.6-6.2)	2.0 (1.3-3.0)	2.0 (1.3-3.1)
**15**	7.3 (4.4-12.0)	4.2 (2.8-6.4)	4.2 (2.8-6.3)	2.0 (1.5-2.6)	2.0 (1.5-2.7)	1.0 (0.7-1.6)	1.0 (0.8-1.4)	1.0 (0.8-1.4)	1.0 (0.7-1.3)	1.0 (0.7-1.3)	6.9 (3.6-13.3)	4.2 (2.5-7.1)	4.0 (2.6-6.2)	2.0 (1.3-3.0)	2.0 (1.3-3.1)
**20**	7.6 (4.5-12.6)	4.3 (2.8-6.5)	4.2 (2.8-6.3)	2.0 (1.5-2.6)	2.0 (1.5-2.7)	1.0 (0.7-1.6)	1.0 (0.8-1.4)	1.0 (0.8-1.4)	1.0 (0.7-1.3)	1.0 (0.7-1.3)	7.2 (3.7-13.9)	4.2 (2.5-7.1)	4.0 (2.6-6.2)	2.0 (1.3-3.0)	2.0 (1.3-3.1)
**30**	7.9 (4.7-13.3)	4.3 (2.8-6.5)	4.2 (2.8-6.3)	2.0 (1.5-2.6)	2.0 (1.5-2.6)	1.1 (0.7-1.6)	1.0 (0.8-1.4)	1.0 (0.8-1.4)	1.0 (0.7-1.3)	1.0 (0.7-1.3)	7.5 (3.8-14.8)	4.2 (2.5-7.1)	4.0 (2.6-6.2)	2.0 (1.3-3.0)	2.0 (1.3-3.0)
**60**	8.3 (4.8-14.2)	4.3 (2.9-6.6)	4.2 (2.8-6.3)	2.0 (1.5-2.6)	2.0 (1.5-2.6)	1.1 (0.7-1.6)	1.0 (0.8-1.4)	1.0 (0.8-1.4)	1.0 (0.7-1.3)	1.0 (0.7-1.3)	7.9 (3.9-15.8)	4.2 (2.5-7.1)	4.0 (2.6-6.2)	2.0 (1.4-3.0)	2.0 (1.4-3.0)

Odds ratios (ORs) by the standard conditional logistic regression (OR_{SCL}), by the Mantel-Haenszel method (OR_{MH}), by the weighting method (OR_G) and their 95% confidence intervals for exposure and OR_{SCL} and OR_G for time-varying confounder estimated in case-crossover studies for cases and time-controls, and OR_{SCL}, OR_{MH}, and OR_G for exposure and OR_{SCL} and OR_G for time-varying confounder in case–time-control studies for Scenario 5. Some of data in this table is also presented in Web Figures 6A (exposure in case-crossover study, cases), 6B (exposure in case–time-control study), and 6C (time-varying confounder in case-crossover study, cases) for selected values of M.

Web Table 7. Odds Ratios (ORs) for Scenarios 6 (with a binary time-varying confounder, RR = 4, and 30% censoring)

	**Case-crossover study, cases**					**Case-crossover study, time-controls**					**Case-time-control study**				
	**Exposure**			**Time-varying confounder**		**Exposure**			**Time-varying confounder**		**Exposure**			**Time-varying confounder**	
M	**OR_{SCL}**	**OR_{MH}**	**OR_G**	**OR_{SCL}**	**OR_G**	**OR_{SCL}**	**OR_{MH}**	**OR_G**	**OR_{SCL}**	**OR_G**	**OR_{SCL}**	**OR_{MH}**	**OR_G**	**OR_{SCL}**	**OR_G**
**1**	6.1 (4.0-9.1)	6.1 (3.5-10.6)	6.1 (4.0-9.1)	2.0 (1.1-3.6)	2.0 (1.1-3.6)	1.5 (1.1-2.0)	1.5 (1.0-2.1)	1.5 (1.1-2.0)	1.0 (0.7-1.5)	1.0 (0.7-1.5)	4.1 (2.5-6.7)	4.1 (2.1-8.1)	4.1 (2.5-6.7)	2.0 (1.0-4.0)	2.0 (1.0-4.0)
**2**	7.2 (4.7-10.9)	5.9 (3.6-9.8)	6.0 (4.0-9.1)	2.0 (1.3-2.9)	2.0 (1.3-3.0)	1.6 (1.1-2.2)	1.4 (1.0-2.0)	1.5 (1.1-2.0)	1.0 (0.7-1.5)	1.0 (0.7-1.5)	4.6 (2.7-7.7)	4.1 (2.2-7.6)	4.1 (2.5-6.5)	1.9 (1.1-3.3)	2.0 (1.1-3.5)
**3**	8.0 (5.1-12.4)	5.9 (3.6-9.8)	6.0 (3.9-9.2)	2.0 (1.4-2.8)	2.0 (1.4-2.9)	1.6 (1.1-2.3)	1.4 (1.0-2.0)	1.5 (1.1-2.0)	1.0 (0.7-1.4)	1.0 (0.7-1.4)	5.0 (2.8-8.6)	4.1 (2.3-7.5)	4.1 (2.5-6.5)	2.0 (1.2-3.2)	2.0 (1.2-3.3)
**4**	8.6 (5.4-13.6)	5.9 (3.7-9.6)	6.0 (3.9-9.3)	2.0 (1.4-2.7)	2.0 (1.4-2.8)	1.6 (1.1-2.4)	1.4 (1.0-2.0)	1.5 (1.1-2.0)	1.0 (0.7-1.4)	1.0 (0.7-1.4)	5.2 (2.9-9.3)	4.1 (2.3-7.4)	4.1 (2.5-6.5)	1.9 (1.2-3.1)	2.0 (1.2-3.2)
**5**	9.1 (5.7-14.5)	6.0 (3.7-9.6)	6.0 (3.9-9.3)	2.0 (1.4-2.7)	2.0 (1.4-2.8)	1.7 (1.1-2.4)	1.4 (1.0-2.0)	1.5 (1.1-2.0)	1.0 (0.7-1.4)	1.0 (0.7-1.4)	5.5 (3.0-9.9)	4.2 (2.3-7.4)	4.1 (2.5-6.5)	2.0 (1.3-3.0)	2.0 (1.2-3.2)
**6**	9.5 (5.9-15.3)	6.0 (3.7-9.6)	6.0 (3.9-9.4)	2.0 (1.4-2.7)	2.0 (1.4-2.8)	1.7 (1.1-2.5)	1.4 (1.0-2.0)	1.5 (1.1-2.0)	1.0 (0.7-1.4)	1.0 (0.7-1.4)	5.7 (3.1-10.3)	4.1 (2.3-7.4)	4.1 (2.5-6.5)	2.0 (1.3-3.0)	2.0 (1.3-3.2)
**10**	10.7 (6.5-17.6)	6.0 (3.8-9.6)	6.0 (3.9-9.4)	2.0 (1.5-2.6)	2.0 (1.5-2.7)	1.7 (1.1-2.6)	1.4 (1.0-2.0)	1.5 (1.1-2.0)	1.0 (0.7-1.4)	1.0 (0.7-1.4)	6.2 (3.3-11.7)	4.2 (2.4-7.3)	4.0 (2.5-6.5)	2.0 (1.3-3.0)	2.0 (1.3-3.1)
**12**	11.1 (6.6-18.5)	6.1 (3.8-9.7)	6.0 (3.8-9.4)	2.0 (1.5-2.6)	2.0 (1.5-2.7)	1.7 (1.1-2.7)	1.5 (1.1-2.0)	1.5 (1.1-2.0)	1.0 (0.7-1.4)	1.0 (0.7-1.4)	6.4 (3.3-12.2)	4.2 (2.4-7.3)	4.0 (2.5-6.5)	2.0 (1.3-3.0)	2.0 (1.3-3.1)
**15**	11.5 (6.8-19.5)	6.1 (3.9-9.7)	6.0 (3.8-9.4)	2.0 (1.5-2.6)	2.0 (1.5-2.7)	1.8 (1.1-2.7)	1.5 (1.1-2.0)	1.5 (1.1-2.0)	1.0 (0.7-1.3)	1.0 (0.7-1.4)	6.6 (3.4-12.8)	4.2 (2.4-7.3)	4.0 (2.5-6.5)	2.0 (1.3-3.0)	2.0 (1.3-3.0)
**20**	12.1 (7.1-20.7)	6.2 (3.9-9.8)	6.0 (3.8-9.5)	2.0 (1.5-2.6)	2.0 (1.5-2.7)	1.8 (1.1-2.7)	1.5 (1.1-2.0)	1.5 (1.1-2.0)	1.0 (0.7-1.3)	1.0 (0.7-1.4)	6.8 (3.5-13.5)	4.2 (2.4-7.3)	4.0 (2.5-6.5)	2.0 (1.3-2.9)	2.0 (1.3-3.0)
**30**	12.7 (7.3-22.2)	6.2 (3.9-9.8)	6.0 (3.8-9.5)	2.0 (1.5-2.6)	2.0 (1.5-2.7)	1.8 (1.1-2.8)	1.5 (1.1-2.0)	1.5 (1.1-2.0)	1.0 (0.7-1.3)	1.0 (0.7-1.3)	7.1 (3.5-14.4)	4.2 (2.4-7.4)	4.0 (2.5-6.5)	2.0 (1.3-2.9)	2.0 (1.3-3.0)
**60**	13.6 (7.6-24.3)	6.3 (4.0-9.9)	6.0 (3.8-9.5)	2.0 (1.5-2.6)	2.0 (1.5-2.6)	1.8 (1.1-2.8)	1.5 (1.1-2.0)	1.5 (1.1-2.0)	1.0 (0.7-1.3)	1.0 (0.7-1.3)	7.5 (3.6-15.6)	4.2 (2.4-7.3)	4.0 (2.5-6.5)	2.0 (1.3-2.9)	2.0 (1.3-3.0)

Odds ratios (ORs) by the standard conditional logistic regression (OR_{SCL}), by the Mantel-Haenszel method (OR_{MH}), by the weighting method (OR_G) and their 95% confidence intervals for exposure and OR_{SCL} and OR_G for time-varying confounder estimated in case-crossover studies for cases and time-controls, and OR_{SCL}, OR_{MH}, and OR_G for exposure and OR_{SCL} and OR_G for time-varying confounder in case–time-control studies for Scenario 6. Some of data in this table is also presented in Web Figures 6D (exposure in case-crossover study, cases), 6E (exposure in case–time-control study), and 6F (time-varying confounder in case-crossover study, cases) for selected values of M.

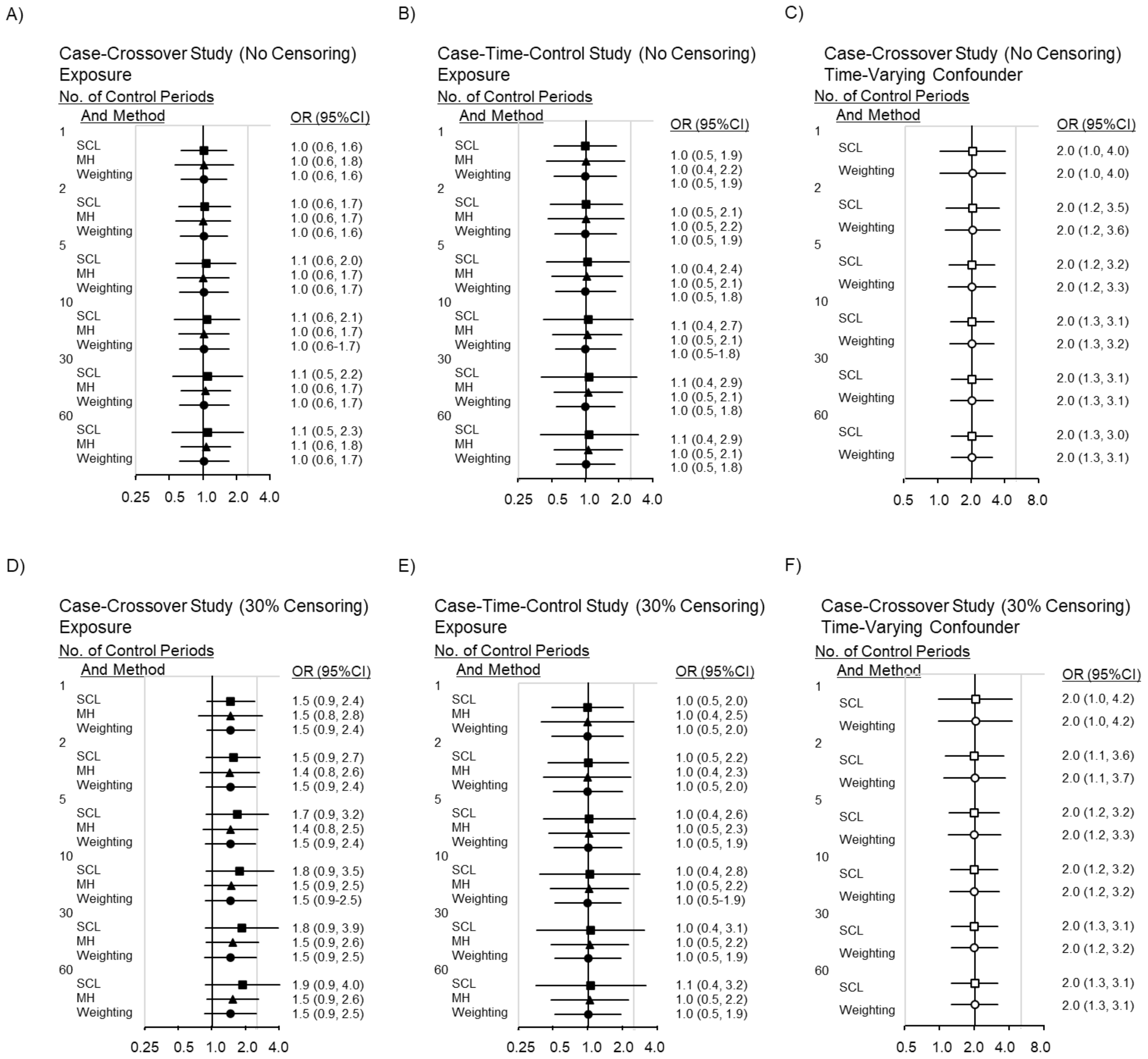

Web Figure 7. Odds ratios (ORs) with 95% CIs estimated in case-crossover studies for exposure and binary time-varying confounder in Scenarios 7 and 8 (RR = 1, RRz = 2)

Web Figures 7A, 7B, and 7C show ORs for exposure in case-crossover studies, exposure in case–time-control studies, and time-varying confounder of cases in case-crossover studies, respectively for Scenario 7 where no patients are censored at the end of the exposed period. Web Figures 7D, 7E, and 7F show ORs for exposure in case-crossover studies, exposure in case–time-control studies, and time-varying confounder of cases in case-crossover studies, respectively for Scenario 8 where 30% of patients are censored at the end of the exposed period.

RR: true rate ratio for exposure; RRz: true rate ratio for time-varying confounder; OR: estimated odds ratio; SCL: OR by the standard conditional logistic regression; MH: OR by the Mantel-Haenszel method; Weighting: OR by the weighting method; CI: confidence interval.

Web Table 8. Odds Ratios (ORs) for Scenarios 7 (with a binary time-varying confounder, RR = 1, and no censoring)

	**Case-crossover study, cases**					**Case-crossover study, time-controls**					**Case-time-control study**				
	**Exposure**			**Time-varying confounder**		**Exposure**			**Time-varying confounder**		**Exposure**			**Time-varying confounder**	
**M**	**OR_{SCL}**	**OR_{MH}**	**OR_{G}**	**OR_{SCL}**	**OR_{G}**	**OR_{SCL}**	**OR_{MH}**	**OR_{G}**	**OR_{SCL}**	**OR_{G}**	**OR_{SCL}**	**OR_{MH}**	**OR_{G}**	**OR_{SCL}**	**OR_{G}**
**1**	1.0 (0.6-1.6)	1.0 (0.6-1.8)	1.0 (0.6-1.6)	2.0 (1.0-4.0)	2.0 (1.0-4.0)	1.0 (0.7-1.6)	1.0 (0.6-1.8)	1.0 (0.7-1.6)	1.0 (0.5-1.9)	1.0 (0.5-1.9)	1.0 (0.5-1.9)	1.0 (0.4-2.2)	1.0 (0.5-1.9)	2.1 (0.8-5.2)	2.1 (0.8-5.2)
**2**	1.0 (0.6-1.7)	1.0 (0.6-1.7)	1.0 (0.6-1.6)	2.0 (1.2-3.5)	2.0 (1.2-3.6)	1.0 (0.6-1.7)	1.0 (0.6-1.7)	1.0 (0.6-1.6)	1.0 (0.6-1.7)	1.0 (0.6-1.8)	1.0 (0.5-2.1)	1.0 (0.5-2.2)	1.0 (0.5-1.9)	2.1 (0.9-4.6)	2.0 (0.9-4.6)
**3**	1.0 (0.6-1.8)	1.0 (0.6-1.7)	1.0 (0.6-1.7)	2.0 (1.2-3.3)	2.0 (1.2-3.4)	1.0 (0.6-1.7)	1.0 (0.6-1.6)	1.0 (0.6-1.6)	1.0 (0.6-1.7)	1.0 (0.6-1.7)	1.0 (0.5-2.2)	1.0 (0.5-2.1)	1.0 (0.5-1.8)	2.0 (1.0-4.3)	2.0 (0.9-4.3)
**4**	1.0 (0.6-1.9)	1.0 (0.6-1.7)	1.0 (0.6-1.7)	2.0 (1.2-3.3)	2.0 (1.2-3.4)	1.0 (0.6-1.8)	1.0 (0.6-1.6)	1.0 (0.6-1.6)	1.0 (0.6-1.7)	1.0 (0.6-1.7)	1.0 (0.5-2.3)	1.0 (0.5-2.1)	1.0 (0.5-1.8)	2.0 (1.0-4.2)	2.0 (1.0-4.3)
**5**	1.1 (0.6-2.0)	1.0 (0.6-1.7)	1.0 (0.6-1.7)	2.0 (1.2-3.2)	2.0 (1.2-3.3)	1.0 (0.6-1.8)	1.0 (0.6-1.6)	1.0 (0.6-1.6)	1.0 (0.6-1.7)	1.0 (0.6-1.7)	1.0 (0.4-2.4)	1.0 (0.5-2.1)	1.0 (0.5-1.8)	2.0 (1.0-4.1)	2.0 (1.0-4.2)
**6**	1.1 (0.6-2.0)	1.0 (0.6-1.7)	1.0 (0.6-1.7)	2.0 (1.3-3.2)	2.0 (1.2-3.2)	1.0 (0.6-1.8)	1.0 (0.6-1.6)	1.0 (0.6-1.6)	1.0 (0.6-1.6)	1.0 (0.6-1.7)	1.0 (0.4-2.5)	1.0 (0.5-2.1)	1.0 (0.5-1.8)	2.0 (1.0-4.1)	2.0 (1.0-4.1)
**10**	1.1 (0.6-2.1)	1.0 (0.6-1.7)	1.0 (0.6-1.7)	2.0 (1.3-3.1)	2.0 (1.3-3.2)	1.0 (0.5-1.9)	1.0 (0.6-1.6)	1.0 (0.6-1.6)	1.0 (0.6-1.6)	1.0 (0.6-1.7)	1.1 (0.4-2.7)	1.0 (0.5-2.1)	1.0 (0.5-1.8)	2.0 (1.0-3.9)	2.0 (1.0-4.0)
**12**	1.1 (0.5-2.1)	1.0 (0.6-1.7)	1.0 (0.6-1.7)	2.0 (1.3-3.1)	2.0 (1.3-3.2)	1.0 (0.5-1.9)	1.0 (0.6-1.6)	1.0 (0.6-1.6)	1.0 (0.6-1.6)	1.0 (0.6-1.6)	1.1 (0.4-2.7)	1.0 (0.5-2.1)	1.0 (0.5-1.8)	2.0 (1.1-3.9)	2.0 (1.0-4.0)
**15**	1.1 (0.5-2.2)	1.0 (0.6-1.7)	1.0 (0.6-1.7)	2.0 (1.3-3.1)	2.0 (1.3-3.1)	1.0 (0.5-2.0)	1.0 (0.6-1.6)	1.0 (0.6-1.6)	1.0 (0.6-1.6)	1.0 (0.6-1.6)	1.1 (0.4-2.8)	1.0 (0.5-2.1)	1.0 (0.5-1.8)	2.0 (1.1-3.9)	2.0 (1.0-4.0)
**20**	1.1 (0.5-2.2)	1.0 (0.6-1.7)	1.0 (0.6-1.7)	2.0 (1.3-3.1)	2.0 (1.3-3.1)	1.0 (0.5-2.0)	1.0 (0.6-1.6)	1.0 (0.6-1.6)	1.0 (0.6-1.6)	1.0 (0.6-1.6)	1.1 (0.4-2.8)	1.0 (0.5-2.1)	1.0 (0.5-1.8)	2.0 (1.1-3.9)	2.0 (1.1-3.9)
**30**	1.1 (0.5-2.2)	1.0 (0.6-1.7)	1.0 (0.6-1.7)	2.0 (1.3-3.1)	2.0 (1.3-3.1)	1.0 (0.5-2.0)	1.0 (0.6-1.6)	1.0 (0.6-1.6)	1.0 (0.6-1.6)	1.0 (0.6-1.6)	1.1 (0.4-2.9)	1.0 (0.5-2.1)	1.0 (0.5-1.8)	2.0 (1.1-3.9)	2.0 (1.1-4.0)
**60**	1.1 (0.5-2.3)	1.1 (0.6-1.8)	1.0 (0.6-1.7)	2.0 (1.3-3.0)	2.0 (1.3-3.1)	1.0 (0.5-2.0)	1.0 (0.6-1.6)	1.0 (0.6-1.6)	1.0 (0.6-1.6)	1.0 (0.6-1.6)	1.1 (0.4-2.9)	1.0 (0.5-2.1)	1.0 (0.5-1.8)	2.0 (1.1-3.8)	2.0 (1.1-3.9)

Odds ratios (ORs) by the standard conditional logistic regression (OR_{SCL}), by the Mantel-Haenszel method (OR_{MH}), by the weighting method (OR_{G}) and their 95% confidence intervals for exposure and OR_{SCL} and OR_{G} for time-varying confounder estimated in case-crossover studies for cases and time-controls, and OR_{SCL}, OR_{MH}, and OR_{G} for exposure and OR_{SCL} and OR_{G} for time-varying confounder in case–time-control studies for Scenario 7. Some of data in this table is also presented in Web Figures 7A (exposure in case-crossover study, cases), 7B (exposure in case–time-control study), and 7C (time-varying confounder in case-crossover study, cases) for selected values of M.

Web Table 9. Odds Ratios (ORs) for Scenarios 8 (with a binary time-varying confounder, RR = 1, and 30% censoring)

	Case-crossover study, cases					Case-crossover study, time-controls					Case-time-control study				
	Exposure			Time-varying confounder		Exposure			Time-varying confounder		Exposure			Time-varying confounder	
M	OR_{SCL}	OR_{MH}	OR_G	OR_{SCL}	OR_G	OR_{SCL}	OR_{MH}	OR_G	OR_{SCL}	OR_G	OR_{SCL}	OR_{MH}	OR_G	OR_{SCL}	OR_G
1	1.5 (0.9-2.4)	1.5 (0.8-2.8)	1.5 (0.9-2.4)	2.0 (1.0-4.2)	2.0 (1.0-4.2)	1.5 (0.9-2.4)	1.5 (0.8-2.8)	1.5 (0.9-2.4)	1.0 (0.5-2.2)	1.0 (0.5-2.2)	1.0 (0.5-2.0)	1.0 (0.4-2.5)	1.0 (0.5-2.0)	2.0 (0.7-5.9)	2.0 (0.7-5.9)
2	1.5 (0.9-2.7)	1.4 (0.8-2.6)	1.5 (0.9-2.4)	2.0 (1.1-3.6)	2.0 (1.1-3.7)	1.5 (0.9-2.7)	1.5 (0.8-2.6)	1.5 (0.9-2.5)	1.0 (0.5-1.9)	1.0 (0.5-1.9)	1.0 (0.5-2.2)	1.0 (0.4-2.3)	1.0 (0.5-2.0)	2.0 (0.9-4.7)	2.0 (0.8-4.9)
3	1.6 (0.9-2.9)	1.4 (0.8-2.6)	1.5 (0.9-2.4)	2.0 (1.2-3.5)	2.0 (1.1-3.5)	1.6 (0.9-2.9)	1.4 (0.8-2.6)	1.5 (0.9-2.5)	1.0 (0.5-1.8)	1.0 (0.5-1.8)	1.0 (0.4-2.3)	1.0 (0.4-2.3)	1.0 (0.5-1.9)	2.0 (0.9-4.5)	2.0 (0.9-4.7)
4	1.6 (0.9-3.1)	1.4 (0.8-2.5)	1.5 (0.9-2.4)	2.0 (1.2-3.3)	2.0 (1.2-3.4)	1.6 (0.9-3.0)	1.4 (0.8-2.6)	1.5 (0.9-2.5)	1.0 (0.6-1.7)	1.0 (0.5-1.8)	1.0 (0.4-2.5)	1.0 (0.4-2.3)	1.0 (0.5-1.9)	2.0 (0.9-4.3)	2.0 (0.9-4.5)
5	1.7 (0.9-3.2)	1.4 (0.8-2.5)	1.5 (0.9-2.4)	2.0 (1.2-3.2)	2.0 (1.2-3.3)	1.6 (0.9-3.1)	1.4 (0.8-2.5)	1.5 (0.9-2.5)	1.0 (0.6-1.7)	1.0 (0.6-1.8)	1.0 (0.4-2.6)	1.0 (0.5-2.3)	1.0 (0.5-1.9)	2.0 (0.9-4.2)	2.0 (0.9-4.4)
6	1.7 (0.9-3.3)	1.4 (0.8-2.5)	1.5 (0.9-2.5)	2.0 (1.2-3.2)	2.0 (1.2-3.3)	1.7 (0.9-3.2)	1.4 (0.8-2.5)	1.5 (0.9-2.5)	1.0 (0.6-1.7)	1.0 (0.6-1.7)	1.0 (0.4-2.6)	1.0 (0.4-2.3)	1.0 (0.5-1.9)	2.0 (1.0-4.2)	2.0 (0.9-4.4)
10	1.8 (0.9-3.5)	1.5 (0.9-2.5)	1.5 (0.9-2.5)	2.0 (1.2-3.2)	2.0 (1.2-3.2)	1.7 (0.8-3.5)	1.4 (0.8-2.5)	1.5 (0.9-2.5)	1.0 (0.6-1.7)	1.0 (0.6-1.7)	1.0 (0.4-2.8)	1.0 (0.5-2.2)	1.0 (0.5-1.9)	2.0 (1.0-4.0)	2.0 (1.0-4.1)
12	1.8 (0.9-3.6)	1.5 (0.9-2.5)	1.5 (0.9-2.5)	2.0 (1.2-3.2)	2.0 (1.2-3.2)	1.7 (0.8-3.5)	1.4 (0.8-2.5)	1.5 (0.9-2.5)	1.0 (0.6-1.7)	1.0 (0.6-1.7)	1.0 (0.4-2.9)	1.0 (0.5-2.2)	1.0 (0.5-1.9)	2.0 (1.0-4.1)	2.0 (1.0-4.2)
15	1.8 (0.9-3.7)	1.5 (0.9-2.6)	1.5 (0.9-2.5)	2.0 (1.2-3.1)	2.0 (1.2-3.2)	1.7 (0.8-3.6)	1.4 (0.8-2.5)	1.5 (0.9-2.5)	1.0 (0.6-1.7)	1.0 (0.6-1.7)	1.0 (0.4-2.9)	1.0 (0.5-2.3)	1.0 (0.5-1.9)	2.0 (1.0-4.0)	2.0 (1.0-4.1)
20	1.8 (0.9-3.8)	1.5 (0.9-2.6)	1.5 (0.9-2.5)	2.0 (1.3-3.1)	2.0 (1.2-3.2)	1.7 (0.8-3.7)	1.5 (0.8-2.5)	1.5 (0.9-2.5)	1.0 (0.6-1.6)	1.0 (0.6-1.7)	1.0 (0.4-3.0)	1.0 (0.5-2.2)	1.0 (0.5-1.9)	2.0 (1.0-4.0)	2.0 (1.0-4.1)
30	1.8 (0.9-3.9)	1.5 (0.9-2.6)	1.5 (0.9-2.5)	2.0 (1.3-3.1)	2.0 (1.2-3.2)	1.8 (0.8-3.8)	1.5 (0.8-2.5)	1.5 (0.9-2.5)	1.0 (0.6-1.6)	1.0 (0.6-1.6)	1.0 (0.4-3.1)	1.0 (0.5-2.2)	1.0 (0.5-1.9)	2.0 (1.0-3.9)	2.0 (1.0-4.0)
60	1.9 (0.9-4.0)	1.5 (0.9-2.6)	1.5 (0.9-2.5)	2.0 (1.3-3.1)	2.0 (1.3-3.1)	1.8 (0.8-3.9)	1.5 (0.9-2.5)	1.5 (0.9-2.6)	1.0 (0.6-1.6)	1.0 (0.6-1.6)	1.1 (0.4-3.2)	1.0 (0.5-2.2)	1.0 (0.5-1.9)	2.0 (1.0-3.9)	2.0 (1.0-4.0)

Odds ratios (ORs) by the standard conditional logistic regression (OR_{SCL}), by the Mantel-Haenszel method (OR_{MH}), by the weighting method (OR_G) and their 95% confidence intervals for exposure and OR_{SCL} and OR_G for time-varying confounder estimated in case-crossover studies for cases and time-controls, and OR_{SCL}, OR_{MH}, and OR_G for exposure and OR_{SCL} and OR_G for time-varying confounder in case–time-control studies for Scenario 8. Some of data in this table is also presented in Web Figures 7D (exposure in case-crossover study, cases), 7E (exposure in case–time-control study), and 7F (time-varying confounder in case-crossover study, cases) for selected values of M.
